# Supplementary material for: History of heart failure and chronic kidney disease and risk of all-cause death after COVID-19 during the first three waves of the pandemic in comparison with influenza outbreaks in Sweden: a registry-based, retrospective, case–control study
Source: BMJ Open. 2023 Apr 28;13(4):e069037. doi: 10.1136/bmjopen-2022-069037 (PMC10151240; doi:10.1136/bmjopen-2022-069037)
Supplement: Supplementary data [file bmjopen-2022-069037supp001.pdf]

Supplemental material

Table 1. Adjusted HR and 95% CI for all-cause death in COVID-19 patients.

	Hazard ratio	95% CI	P-value
Cardiorenal disease (CRD) vs. no CRD	1.41	(1.36-1.46)	<0.001
Age per 10 years increase	1.61	(1.58-1.64)	<0.001
Female sex	0.76	(0.73-0.78)	<0.001
Diabetes	1.13	(1.09-1.17)	<0.001
Coronary artery disease	1.00	(0.97-1.03)	0.881
Stroke	1.11	(1.07-1.15)	<0.001
Peripheral artery disease (PAD)	1.25	(1.19-1.31)	<0.001
Pneumonia	1.19	(1.16-1.23)	<0.001
Chronic obstructive pulmonary disease (COPD)	1.08	(1.04-1.13)	<0.001
Obesity	0.97	(0.92-1.03)	0.335
Rheumatologic disease	0.99	(0.95-1.03)	0.570
D-vitamin deficiency	1.10	(1.06-1.15)	<0.001
RAAS inhibitors	0.86	(0.83-0.89)	<0.001
Paracetamol	1.21	(1.17-1.25)	<0.001
Corticosteroids	1.18	(1.14-1.22)	<0.001
Statins	0.82	(0.79-0.85)	<0.001

Table 2. Adjusted HR and 95% CI for all-cause death in COVID-19 excluding patients with ZV100 as a main diagnosis.

	Hazard ratio	95% CI	P-value
Cardiorenal disease (CRD) vs. no CRD	1.43	(1.35-1.51)	<0.001
Age per 10 years increase	1.70	(1.65-1.75)	<0.001
Female sex	0.77	(0.73-0.81)	<0.001
Diabetes	1.17	(1.11-1.23)	<0.001
Coronary artery disease	1.03	(0.98-1.08)	0.315
Stroke	1.17	(1.11-1.23)	<0.001
Peripheral artery disease (PAD)	1.35	(1.25-1.45)	<0.001
Pneumonia	1.19	(1.13-1.25)	<0.001
Chronic obstructive pulmonary disease (COPD)	1.07	(1.01-1.14)	0.033
Obesity	0.98	(0.89-1.07)	0.601
Rheumatologic disease	1.06	(0.99-1.13)	0.071
D-vitamin deficiency	1.09	(1.02-1.16)	0.008
RAAS inhibitors	0.88	(0.84-0.93)	<0.001
Paracetamol	1.23	(1.17-1.30)	<0.001
Corticosteroids	1.13	(1.07-1.19)	<0.001
Statins	0.82	(0.78-0.87)	<0.001

Table 3. Baseline characteristics in men with COVID-19 or influenza with and without cardiorenal disease (CRD).

	Covid-19 with CRD n=16721	Covid-19 without CRD n=8670	Influenza with CRD n=3283	Influenza without CRD n=1638
Age, years, mean (SD)	77.7 (11.7)	80.1 (10.4)	78.1 (11.9)	82.1 (9.1)
Age, years, median (min-max)	79 (18-103)	82 (20-103)	80 (20-106)	83 (24-104)
Females, n (%)	0 (0)	0 (0)	0 (0)	0 (0)
Year				
2020, n (%)	11329 (68)	5477 (63)	0 (0)	0 (0)
2021, n (%)	5392 (32)	3193 (37)	0 (0)	0 (0)
2015, n (%)	0 (0)	0 (0)	515 (16)	246 (15)
2016, n (%)	0 (0)	0 (0)	459 (14)	227 (14)
2017, n (%)	0 (0)	0 (0)	624 (19)	328 (20)
2018, n (%)	0 (0)	0 (0)	1051 (32)	531 (32)
2019, n (%)	0 (0)	0 (0)	634 (19)	306 (19)
In-hospital stay, days, median (IQR)	6 (4-11)	6 (4-10)	6 (4-10)	5 (3-9)
Heart failure, n (%)	13275 (79)	0 (0)	2648 (81)	0 (0)
Chronic kidney disease (CKD), n (%)	7025 (42)	0 (0)	1260 (38)	0 (0)
Dialysis, n (%)	1174 (7)	0 (0)	350 (11)	0 (0)
Coronary artery disease, n (%)	8810 (53)	2270 (26)	1882 (57)	485 (30)
Stroke, n (%)	4747 (28)	2141 (25)	967 (29)	441 (27)
Atrial fibrillation, n (%)	9102 (54)	1904 (22)	1759 (54)	379 (23)
Peripheral artery disease (PAD), n (%)	2127 (13)	488 (6)	422 (13)	86 (5)
Diabetes, n (%)	6158 (37)	1863 (21)	1104 (34)	372 (23)
Chronic obstructive pulmonary disease (COPD), n (%)	3243 (19)	772 (9)	688 (21)	161 (10)
Any history of pneumonia (viral/bacterial), n (%)	6276 (38)	1681 (19)	1519 (46)	475 (29)
Any history of thromboembolism (DVT or PE), n (%)	2120 (13)	741 (9)	399 (12)	139 (8)
Vitamin-D deficiency, n (%)	3081 (18)	450 (5)	610 (19)	70 (4)
Cancer, n (%)	6372 (38)	3300 (38)	1175 (36)	581 (35)
Any of ACEi/ARB/MRA/ARNi/SGLT2i/β-blocker, n (%)*	15056 (90)	5322 (61)	2974 (91)	1023 (62)
RAAS inhibitor, n (%)	11293 (68)	4027 (46)	2307 (70)	729 (45)
Statins, n (%)	9733 (58)	3521 (41)	1875 (57)	655 (40)
Corticosteroids, n (%)	4585 (27)	1492 (17)	1081 (33)	339 (21)
Paracetamol, n (%)	8850 (53)	3497 (40)	1667 (51)	656 (40)
Modulating antineoplastic and hormone drugs, n (%)	1182 (7)	706 (8)	210 (6)	110 (7)

* Any of Angiotensin-converting enzyme (ACE) inhibitors, Angiotensin II receptor blockers (ARBs), Mineralocorticoid receptor antagonists (MRA), Angiotensin receptor-neprilysin inhibitors (ARNi), Sodium-glucose Cotransporter-2 (SGLT2) inhibitors and Beta blockers.

Table 4. Baseline characteristics in women with COVID-19 or influenza with and without cardiorenal disease (CRD).

	Covid-19 with CRD n=12474	Covid-19 without CRD n=7001	Influenza with CRD n=2700	Influenza without CRD n=1276
Age, years, mean (SD)	80.9 (12.0)	82.6 (12.3)	81.1 (12.4)	84.3 (9.6)
Age, years, median (min-max)	83 (18-107)	85 (18-106)	84 (20-102)	86 (32-107)
Females, n (%)	12474 (100)	7001 (100)	2700 (100)	1276 (100)
Year				
2020, n (%)	8400 (67)	4437 (63)	0 (0)	0 (0)
2021, n (%)	4074 (33)	2564 (37)	0 (0)	0 (0)
2015, n (%)	0 (0)	0 (0)	431 (16)	197 (15)
2016, n (%)	0 (0)	0 (0)	402 (15)	147 (12)
2017, n (%)	0 (0)	0 (0)	530 (20)	276 (22)
2018, n (%)	0 (0)	0 (0)	839 (31)	432 (34)
2019, n (%)	0 (0)	0 (0)	498 (18)	224 (18)
In-hospital stay, days, median (IQR)	7 (4-11)	6 (4-10)	7 (4-11)	6 (4-11)
Heart failure, n (%)	10374 (83)	0 (0)	2323 (86)	0 (0)
Chronic kidney disease (CKD), n (%)	4288 (34)	0 (0)	766 (28)	0 (0)
Dialysis, n (%)	603 (5)	0 (0)	170 (6)	0 (0)
Coronary artery disease, n (%)	5504 (44)	1335 (19)	1311 (49)	301 (24)
Stroke, n (%)	3402 (27)	1514 (22)	724 (27)	291 (23)
Atrial fibrillation, n (%)	6415 (51)	1274 (18)	1418 (53)	266 (21)
Peripheral artery disease (PAD), n (%)	1440 (12)	331 (5)	259 (10)	72 (6)
Diabetes, n (%)	3892 (31)	1111 (16)	739 (27)	208 (16)
Chronic obstructive pulmonary disease (COPD), n (%)	2839 (23)	649 (9)	649 (24)	163 (13)
Any history of pneumonia (viral/bacterial), n (%)	4799 (38)	1256 (18)	1270 (47)	380 (30)
Any history of thromboembolism (DVT or PE), n (%)	1824 (15)	666 (10)	393 (15)	131 (10)
Vitamin-D deficiency, n (%)	2699 (22)	664 (9)	474 (18)	86 (7)
Cancer, n (%)	4577 (37)	2480 (35)	906 (34)	433 (34)
Any of ACEi/ARB/MRA/ARNi/SGLT2i/β-blocker, n (%)*	11131 (89)	4355 (62)	2427 (90)	840 (66)
RAAS inhibitor, n (%)	7992 (64)	3095 (44)	1766 (65)	570 (45)
Statins, n (%)	5437 (44)	2045 (29)	1059 (39)	373 (29)
Corticosteroids, n (%)	3927 (31)	1418 (20)	1015 (38)	360 (28)
Paracetamol, n (%)	8466 (68)	3828 (55)	1828 (68)	728 (57)
Modulating antineoplastic and hormone drugs, n (%)	516 (4)	256 (4)	89 (3)	35 (3)

* Any of Angiotensin-converting enzyme (ACE) inhibitors, Angiotensin II receptor blockers (ARBs), Mineralocorticoid receptor antagonists (MRA), Angiotensin receptor-neprilysin inhibitors (ARNi), Sodium-glucose Cotransporter-2 (SGLT2) inhibitors and Beta blockers.

Ritsinger V, *et al. BMJ Open* 2024; 13:e069037. doi: 10.1136/bmjopen-2022-069037
