# Supplementary material for: History of heart failure and chronic kidney disease and risk of all-cause death after COVID-19 during the first three waves of the pandemic in comparison with influenza outbreaks in Sweden: a registry-based, retrospective, case–control study
Source: BMJ Open. 2023 Apr 28;13(4):e069037. doi: 10.1136/bmjopen-2022-069037 (PMC10151240; doi:10.1136/bmjopen-2022-069037)
Supplement: Author's manuscript [file bmjopen-2022-069037.draft_revisions.pdf]

BMJ Open

BMJ Open is committed to open peer review. As part of this commitment we make the peer review history of every article we publish publicly available.

When an article is published we post the peer reviewers' comments and the authors' responses online. We also post the versions of the paper that were used during peer review. These are the versions that the peer review comments apply to.

The versions of the paper that follow are the versions that were submitted during the peer review process. They are not the versions of record or the final published versions. They should not be cited or distributed as the published version of this manuscript.

BMJ Open is an open access journal and the full, final, typeset and author-corrected version of record of the manuscript is available on our site with no access controls, subscription charges or pay-per-view fees (http://bmjopen.bmj.com).

If you have any questions on BMJ Open's open peer review process please email info.bmjopen@bmj.com

BMJ Open

History of heart failure and chronic kidney disease and the risk for all-cause death after COVID-19 during the three first waves in comparison to influenza outbreaks in Sweden.

Journal:	*BMJ Open*
Manuscript ID	bmjopen-2022-069037
Article Type:	Original research
Date Submitted by the Author:	14-Oct-2022
Complete List of Authors:	Ritsinger, Viveca; Karolinska Institute Department of Medicine Solna, Department of Medicine; Solna Karolinska Institute, Department of Medicine<br>Bodegård, Johan; AstraZeneca PLC<br>Kristofi, Robin; Uppsala University, Department of Medical Sciences, Clinical Diabetes and Metabolism<br>Thuresson, Marcus; Statisiticon<br>Nathanson, David; Karolinska Institutet, Department of Medicine<br>Nyström, Thomas; Södersjukhuset, Department of Clinical Science and Education<br>Eriksson, Jan; Uppsala University, Department of Medical Sciences, Clinical Diabetes and Metabolism<br>Norhammar, Anna; Karolinska Institute, Division of Medicine MedS, K2;
Keywords:	COVID-19, Heart failure < CARDIOLOGY, EPIDEMIOLOGY, Chronic renal failure < NEPHROLOGY

SCHOLARONE™
Manuscripts

I, the Submitting Author has the right to grant and does grant on behalf of all authors of the Work (as defined in the below author licence), an exclusive licence and/or a non-exclusive licence for contributions from authors who are: i) UK Crown employees; ii) where BMJ has agreed a CC-BY licence shall apply, and/or iii) in accordance with the terms applicable for US Federal Government officers or employees acting as part of their official duties; on a worldwide, perpetual, irrevocable, royalty-free basis to BMJ Publishing Group Ltd ("BMJ") its licensees and where the relevant Journal is co-owned by BMJ to the co-owners of the Journal, to publish the Work in this journal and any other BMJ products and to exploit all rights, as set out in our licence.

The Submitting Author accepts and understands that any supply made under these terms is made by BMJ to the Submitting Author unless you are acting as an employee on behalf of your employer or a postgraduate student of an affiliated institution which is paying any applicable article publishing charge ("APC") for Open Access articles. Where the Submitting Author wishes to make the Work available on an Open Access basis (and intends to pay the relevant APC), the terms of reuse of such Open Access shall be governed by a Creative Commons licence – details of these licences and which Creative Commons licence will apply to this Work are set out in our licence referred to above.

Other than as permitted in any relevant BMJ Author's Self Archiving Policies, I confirm this Work has not been accepted for publication elsewhere, is not being considered for publication elsewhere and does not duplicate material already published. I confirm all authors consent to publication of this Work and authorise the granting of this licence.

For peer review only

History of heart failure and chronic kidney disease and the risk for all-cause death after COVID-19 during the three first waves in comparison to influenza outbreaks in Sweden.

Short title: Heart failure, chronic kidney disease and COVID-19

Authors:

Viveca Ritsinger MD PhD[1, 2], Johan Bodegard MD PhD[3], Robin Kristófi MD[4], Marcus Thuresson PhD[5], David Nathanson MD PhD[6], Thomas Nyström MD PhD[7], Jan W Eriksson MD PhD[4], Anna Norhammar MD PhD[1, 8]

1. Cardiology Unit, Department of Medicine, Solna, Karolinska Institute, Stockholm, Sweden.
2. Department of Research and Development, Region Kronoberg, Växjö, Sweden.
3. Cardiovascular, Renal and Metabolism, Medical Department, BioPharmaceuticals, AstraZeneca Nordic, Oslo, Norway.
4. Department of Medical Sciences, Clinical Diabetes and Metabolism, Uppsala University, Uppsala, Sweden.
5. Statisticon AB, Uppsala, Sweden.
6. Department of Medicine, Huddinge, Karolinska Institutet, Stockholm, Sweden.
7. Department of Clinical Science and Education, Karolinska Institutet, Södersjukhuset, Stockholm, Sweden.
8. Capio S:t Göran Hospital, Stockholm, Sweden.

For peer review only

Abstract: 250 words

Manuscript: 3881 words

Tables: 2

Figures: 5

Corresponding author

Viveca Ritsinger, MD PhD

Cardiology Unit, Department of Medicine

Karolinska Institute, Stockholm, Sweden

E-mail: viveca.ritsinger@ki.se

For peer review only - http://bmjopen.bmj.com/site/about/guidelines.xhtml

Abstract (247)

Objective: To explore how cardiorenal disease (CRD; heart failure and/or chronic kidney disease) impacted mortality in patients hospitalised for COVID-19 during the three first waves in Sweden in comparison to previous influenza outbreaks and with a sex perspective.

Methods: All patients in Sweden with a main hospital diagnosis of COVID-19 (January 2020-September 2021) or influenza (January 2015-December 2019) with previous CRD were identified in registries and compared with a reference group free from CRD but with COVID-19 or influenza (case-control study). Associated risk of all-cause death during the first year was analysed using adjusted Cox proportional hazards models.

Results: In COVID-19 patients with and without prior history of CRD (n=44 866) mean age was 79.8 years (SD 11.8) and 43% were women. In influenza patients (n=8897) mean age was 80.6 (SD 11.5) years and 45% were women. COVID-19 versus influenza was associated with higher mortality risk the first two waves (HR 1.53;95% CI 1.45-1.62,p<0.001 and 1.52;1.44-1.61,p<0.001) but not in the third wave (1.07;0.99-1.14,p=0.072). CRD was an independent risk factor for all-cause death after COVID-19 in men and women (men:1.37;1.31-1.44,p<0.001, women:1.46;1.38-1.54,p<0.001). At ages <70 years women with CRD had a similar mortality rate as men with CRD while at ages ≥70 years mortality rate was higher in men.

Conclusions: Outcome after COVID-19 is worse if CRD is present. In women at ages <70 years the presence of CRD attenuates the protective effect of female sex. Further COVID-19 was associated with higher mortality risk than influenza during the first two waves.

For peer review only - http://bmjopen.bmj.com/site/about/guidelines.xhtml

What is already known on this topic

Previous studies have described an increased in-hospital mortality after COVID-19 hospitalisation compared to that of influenza and identified heart failure and kidney failure in separate publications as risk factors for in-hospital mortality or short-term mortality (four months) in selected patients with COVID-19. To the best of our knowledge few, if any, has made the comparison of mortality between COVID-19 and influenza in the different COVID-19 waves and in patients with cardiorenal disease.

What this study adds

COVID-19 was associated with higher mortality risk than influenza during the first two waves but not in the third. Furthermore, that outcome in patients with COVID-19 and influenza is worse if prior cardiorenal disease is present independently of age and sex. In addition, the presence of cardiorenal disease attenuated the otherwise favourable effect of female sex at ages below 70 years with equal mortality risk in men and women if CRD is present.

How this study might affect research, practice or policy

This study highlights the role of heart failure and chronic kidney disease as independent risk factors for mortality during the first year after COVID-19 and is also a risk factor for mortality after influenza supporting the importance of preventing heart failure and chronic kidney disease in the general population to reduce mortality from severe infections in the future.

For peer review only

For peer review only - http://bmjopen.bmj.com/site/about/guidelines.xhtml

Introduction

Infection with SARS-CoV-2 (COVID-19) affects people globally differently with some individuals more severely affected in need of hospitalisation and in more unfortunate circumstances, causing early mortality and reduced life expectancy. Starting rapidly during the first months after the initial reports from the outbreak in China[1,2], and thereafter, there are multiple reports on COVID-19 disease course and consequences, treatment aspects, pathophysiology and characteristics of high-risk individuals. Early on it was reported that risk factors associated with hospitalisation and mortality from COVID-19 were characteristics such as advanced age, male sex, hypertension, cardiovascular disease, chronic kidney disease (CKD) and obesity.[3-7] Recently asthma and Down's syndrome were added to this list[8], while diabetes has been reported as a risk factor in some studies, while other studies found this risk to be attributed to other comorbidities.[9-12] Due to the fact that co-existence of risk factors and multiple diseases often clusters at higher ages, earlier reports due to low numbers and limited information, could not clarify independent associations. Some reports have been further limited to data on patients with COVID-19 admitted to intensive care, thus possibly introducing selection bias due to aspects on national and local resources and recommendations for such care, including only individuals judged to be in favour of intensive care.[7] Therefore, there is a need of more extensive and deeper analysis on contributing risk factors for severe COVID-19 outcome in a less selected cohort to enhance present knowledge on important risk factors. Such information is of importance for improving future preventive measures. In the present study we aimed to explore how history of heart failure and CKD (i.e., diseases that are notably interrelated and with high risk of cardiovascular and all-cause death[13]), impacts the course from COVID-19 infection and subsequent mortality during the three first waves of the COVID-19 in Sweden, the only western country that did not apply a

For peer review only - http://bmjopen.bmj.com/site/about/guidelines.xhtml

lockdown during the first pandemic year. Further, we aimed to explore this risk in comparison to previous influenza outbreaks and with a sex perspective.

Material and methods

Sources

Sweden has a comprehensive, nationwide public healthcare system. All citizens have a unique personal identification number (person-ID), which is mandatory for all administrative purposes (including any contact with the healthcare system and drug dispensaries), thus providing a complete medical history from a population perspective. This study included data from Statistics Sweden, National Prescribed Drug Register, the National Cause of Death Register and the National Patient Register covering all hospitalisations with discharge diagnoses and all out-patient hospital visits. Individual patient-level data from the national registers were linked using the person-ID by the National Board of Health and Welfare. The linked anonymised database was managed separately by Statisticon AB, Uppsala, Sweden.

COVID-19 and influenza population

The study population includes all patients with CRD from the above specified CARE-19 cohort with a main inpatient hospital diagnosis of COVID-19 (January 1, 2020 to September 9, 2021) or influenza (January 1, 2015 - December 31, 2019). Thus, the study population includes all CRD patients hospitalised for COVID-19 in Sweden and around 2/3 of the total hospitalisations for COVID-19. Of note, the reference group is not chosen based on the COVID-19 or influenza date but on the CRD date as described below.

Study population

For peer review only - http://bmjopen.bmj.com/site/about/guidelines.xhtml

All patients from the CARE-19 cohort above 18 years of age with a main inpatient hospital diagnosis of COVID-19 (January 1, 2020 to September 9, 2021) or influenza (January 1, 2015 - December 31, 2019) were identified in the National Patient Register.

COVID-19 and influenza hospitalised patients were identified with any of the following codes as main diagnosis: U07.1, U07.2, ZV100, J20.8, U04.9, B34.2, B97.2 or J10-J11, J09.9. Furthermore, patients are only included once in each group, at the first COVID-19 or influenza hospitalisation. However, a prior influenza hospitalisation did not exclude the patient if later hospitalised with COVID-19.

CARE-19 cohort database

This database includes all patients hospitalised with heart failure and/or CKD as a main diagnosis recorded in national registries in Sweden since 1995 to September 9, 2021 that also were alive on January 1, 2010. For these patients a reference group without CRD was collected. This reference group was matched by age and sex with cases at the time point for CRD if diagnosed between the years 2010-2020, if diagnosed before 2010 then January 1, 2010 was used as time point for collecting references. Patients with heart failure were defined having at least one hospital diagnosis of heart failure: ICD-10 code I50, I11.0, I13.0, I13.2 and patients with CKD were defined as having at least one hospital diagnosis of CKD: N17-N19, I12.0-I12.9, I13.1, I13.2, N08.3, E10.2, E11.2, E12.2, E13.2, E14.2, Z49, Z99.2.

Patient and public involvement

Patients or the public were not involved in the design, conduct, reporting or dissemination plans of the research.

Study period

For peer review only - http://bmjopen.bmj.com/site/about/guidelines.xhtml

Index date was defined as the date of COVID-19 or influenza hospital admission, which is the reference date for all analyses. Baseline comorbidities were searched based on all hospital data from 1997 when ICD-10 was introduced until the day prior to index, and baseline medications were searched during one year prior to index date until the day prior to index.

Follow-up for events started at the index date (including) and ended at one year, or at date of death or end of follow-up defined as December 31, 2019 for influenza patients and September 9, 2021 for COVID-19 patients.

Statistical methods

Description of baseline characteristics was performed using mean and standard deviation for numerical variables, except for duration of first hospital stay which is described as median and interquartile range. Categorical variables are presented as number and percent. Baseline tables are stratified by type of diagnosis (COVID-19 or influenza) and CRD status (CRD/without CRD). The cumulative risk of all-cause death is presented in Kaplan-Meier curves stratified by type of diagnosis (COVID-19 or influenza) and CRD status (CRD/without CRD).

The relative risk of all-cause death was analysed using a multivariable Cox regression model where the model in addition to COVID-19 vs. influenza and CRD vs. no CRD also included the following variables; age, sex, use of renin-angiotensin system inhibitors (RAAS inhibitors), coronary artery disease, stroke, peripheral artery disease (PAD), diabetes, pneumonia, chronic obstructive pulmonary disease, vitamin D-deficiency, corticosteroids, paracetamol and diagnosed obesity.

To explore the importance of risk factors relative to sex, separate analyses were in addition performed within men and women and by age group.

For peer review only - http://bmjopen.bmj.com/site/about/guidelines.xhtml

An exploratory analysis was performed to evaluate if the risk of all-cause death has changed during the cause of the COVID-19 pandemic period where the COVID-19 inclusion period was split into three-time intervals separated when the frequency of hospitalisations was low. Thus, the time intervals were intended to capture the first three waves of the pandemic in Sweden. The time intervals used: Wave 1: Jan 1, 2020 to August 31, 2020; Wave 2: September 1, 2020 to January 31, 2021 and Wave 3: February 1, 2021 to September 9, 2021. To limit complexity, the analysis was only performed within the CRD diagnosed patients. The cumulative incidence of all-cause death by wave are presented in Kaplan-Meier curves with Influenza as reference. All output from the statistical models is presented with 95 % confidence intervals and p-values. No adjustment for multiplicity has been performed.

Results

Characteristics in COVID-19 patients with heart failure and/or CKD

In all, 44 866 patients hospitalised with a main diagnosis of COVID-19 were identified. Of those, 23 649 had a prior history of heart failure, 11 313 had a prior history of CKD, 5767 had a prior history of the combination of heart failure and CKD and 15 671 references were free from previous CRD.

Baseline characteristics for patients are presented in Table 1.

For peer review only - http://bmjopen.bmj.com/site/about/guidelines.xhtml

Table 1. Baseline characteristics in patients with COVID-19 or influenza with and without cardiorenal disease (CRD).

	COVID-19 with CRD n=29195	COVID-19 without CRD n=15671	Influenza with CRD n=5983	Influenza without CRD n=2914
Age, years, mean (SD)	79.0 (11.9)	81.2 (11.3)	79.5 (12.3)	83.1 (9.4)
Age, years, median (SD)	81 (18-107)	83 (18-106)	82 (20-106)	84 (24-107)
Females, n (%)	12474 (43)	7001 (45)	2700 (45)	1276 (44)
Year				
2020, n (%)	19729 (68)	9914 (63)	0 (0)	0 (0)
2021, n (%)	9466 (32)	5757 (37)	0 (0)	0 (0)
2015, n (%)	0 (0)	0 (0)	946 (16)	443 (15)
2016, n (%)	0 (0)	0 (0)	861 (14)	374 (13)
2017, n (%)	0 (0)	0 (0)	1154 (19)	604 (21)
2018, n (%)	0 (0)	0 (0)	1890 (32)	963 (33)
2019, n (%)	0 (0)	0 (0)	1132 (19)	530 (18)
In-hospital stay, days, median (IQR)	6 (4-11)	6 (4-10)	6 (4-10)	6 (4-10)
Heart failure, n (%)	23649 (81)	0 (0)	4971 (83)	0 (0)
Chronic kidney disease (CKD), n (%)	11313 (39)	0 (0)	2026 (34)	0 (0)
Dialysis, n (%)	1777 (6)	0 (0)	520 (9)	0 (0)
Coronary artery disease, n (%)	14314 (49)	3605 (23)	3193 (53)	786 (27)
Stroke, n (%)	8149 (28)	3655 (23)	1691 (28)	732 (25)
Atrial fibrillation, n (%)	15517 (53)	3178 (20)	3177 (53)	645 (22)
Peripheral artery disease, n (%)	3567 (12)	819 (5)	681 (11)	158 (5)
Diabetes, n (%)	10050 (34)	2974 (19)	1843 (31)	580 (20)
Chronic obstructive pulmonary disease (COPD), n (%)	6082 (21)	1421 (9)	1337 (22)	324 (11)
Any history of pneumonia (viral/bacterial), n (%)	11075 (38)	2937 (19)	2789 (47)	855 (29)
Any history of thromboembolism (DVT or PE), n (%)	3944 (14)	1407 (9)	792 (13)	270 (9)
Vitamin-D deficiency, n (%)	5780 (20)	1114 (7)	1084 (18)	156 (5)
Cancer, n (%)	10949 (38)	5780 (37)	2081 (35)	1014 (35)
Any of ACEi/ARB/MRA/ARNi/SGLT2i/β-blocker, n (%)*	26187 (90)	9677 (62)	5401 (90)	1863 (64)
RAAS inhibitor, n (%)	19285 (66)	7122 (45)	4073 (68)	1299 (45)
Statins, n (%)	15170 (52)	5566 (36)	2934 (49)	1028 (35)
Corticosteroids, n (%)	8512 (29)	2910 (19)	2096 (35)	699 (24)
Paracetamol, n (%)	17316 (59)	7325 (47)	3495 (58)	1384 (47)
Modulating antineoplastic and hormone drugs, n (%)	1698 (6)	962 (6)	299 (5)	145 (5)

* Any of Angiotensin-converting enzyme (ACE) inhibitors, Angiotensin II receptor blockers (ARBs), Mineralocorticoid receptor antagonists (MRA), Angiotensin receptor-neprilysin inhibitors (ARNi), Sodium-glucose Cotransporter-2 (SGLT2) inhibitors and Beta blockers.

Patients with COVID-19 and CRD also had other comorbidities as atrial fibrillation 53%, coronary artery disease 49%, cancer 38%, diabetes 34%, previous pneumonia 38%, stroke

For peer review only - http://bmjopen.bmj.com/site/about/guidelines.xhtml

28%, chronic obstructive pulmonary disease 21%, vitamin D deficiency 20%, history of thromboembolism 14% and peripheral artery disease 12%. Cardiovascular treatment prior to hospitalisation was commonly used with heart failure drug treatment in 90% (any of Angiotensin-converting enzyme [ACE] inhibitors, Angiotensin II receptor blockers [ARBs], Mineralocorticoid receptor antagonists [MRA], Angiotensin receptor-neprilysin inhibitors [ARNi], Sodium-glucose Cotransporter-2 [SGLT2] inhibitors and Beta blockers). Statins were used in 52% and corticosteroids in 29%.

Characteristics in COVID-19 patients without heart failure and/or CKD (reference group)

In contrary, in the reference group (COVID-19 without any history of heart failure or CKD) mean age was 81.2 years (SD 11.3) and 45% were women. Except for cancer the burden of other comorbidities was less compared to COVID-19 patients with CRD.

Characteristics in patients with influenza with heart failure and/or CKD

N=5983 patients with a history of CRD were hospitalised for influenza. Their mean age was 79.5 (SD 12.3) years and 45% were women. Apart from a previous history of pneumonia, reported in 47% in influenza patients with CRD compared to 38% in COVID-19 patients without CRD, patients hospitalised for influenza and CRD the years before the COVID-19 pandemic had a very similar comorbidity pattern as COVID-19 patients with CRD.

Outcome

During a mean follow-up period of 6.9 months (SD 4.3), 16% (n=7085) of the patients with COVID-19 died. All-cause death rate (death per 100 person years) for patients with COVID-

For peer review only - http://bmjopen.bmj.com/site/about/guidelines.xhtml

For peer review only

19 and CRD was 245 compared to 196 in references without CRD. Corresponding mortality rate for influenza between the years 2015-2019 was lower (119 in those with CRD vs. 104 in those without).

Details on cumulative event rates for all-cause death in patients with COVID-19 or influenza stratified by previous CRD are presented in Figure 1. A higher death rate was seen for patients with CRD compared with references without with the highest rate in CRD patients with COVID-19. Except for CRD (1.41; 1.36-1.46, $p<0.001$), other risk factors associated with all-cause death in patients with COVID-19 were age (1.61; 1.58-1.64, $p<0.001$), previous pneumonia (1.19; 1.16-1.23, $p<0.001$) and diabetes (1.13; 1.09-1.17, $p<0.001$; Supplemental Table 1). Female sex was associated with a reduced risk of all-cause death (0.76; 0.73-0.78, $p<0.001$) while no association was seen for coronary artery disease (1.00; 0.97-1.03, $p=0.881$). In an adjusted Cox regression analysis in the total study cohort COVID-19 vs. influenza was associated with an increased risk of all-cause death (HR 1.54; 95% CI 1.47-1.61, $p<0.001$).

Figure 2 depicts cumulative incidence of all-cause death within patients with CRD by the three COVID-19 waves where a similar event rate was seen in the first two waves but with a significantly lower death rate in the third wave although still higher than that observed for influenza. Compared to influenza, COVID-19 was associated with an increased mortality risk in the first two waves (1.53; 1.45-1.62, $p<0.001$ and 1.52; 1.44-1.61, $p<0.001$) but not in the third wave (1.07; 0.99-1.14, $p=0.072$).

Sex specific analyses in patients with COVID-19 and influenza with CRD

Baseline characteristics for men and women are depicted in Supplemental Table 2 and 3. Mean age in men was 77.7 years (SD 11.7) and 80.9 years (SD 12.0) in women with even

For peer review only - http://bmjopen.bmj.com/site/about/guidelines.xhtml

higher mean age in references without previous CRD (men 80.1 [SD 10.4] and women 82.6 years [SD 12.3]). Mean age in influenza patients with CRD was 78.1 (SD 11.9) years in men and 81.1 (SD 12.4) years in women.

Comorbidities in COVID-19 and influenza was equally distributed among men and women apart from coronary artery disease which was more common among men with COVID-19 and CRD (53% vs. 44% in women). Corresponding figures for influenza and CRD was 57% for men vs. 49% in women.

When analysing the cumulative incidence of all-cause death in men (Figure 3A) and women (Figure 3B) there was no major difference. In addition, predictors for a fatal outcome with COVID-19 (illustrated in a Forest plot; Figure 4) were similar in both sexes with the strongest association for age (10-year increase, 1.66; 1.62-1.70, $p<0.001$ for men vs. 1.55; 1.51-1.60, $p<0.001$ for women), CRD (1.37; 1.31-1.44, $p<0.001$ in men vs. 1.46; 1.38-1.54, $p<0.001$ in women), PAD (1.23; 1.15-1.30, $p<0.001$ in men vs. 1.30; 1.21-1.40, $p<0.001$ in women), diabetes (1.10; 1.05-1.15, $p<0.001$ in men vs. 1.18; 1.11-1.24, $p<0.001$ in women) and previous pneumonia (1.19; 1.14-1.25, $p<0.001$ in men vs. 1.19; 1.13-1.25, $p<0.001$ in women). Previous treatment for hypertension and coronary artery disease, such as statins and RAAS inhibitors, were associated with reduced risk for all-cause death in both sexes.

Impact of age and sex

When analysing all-cause death rate in men and women in specified age groups (<60 years, 60-69 years, 70-79 years, ≥80 years) CRD vs. no CRD was associated with a consistent increased mortality rate after COVID-19 independent of sex and age (Figure 5). Men had higher mortality rate than women regardless of CRD, apart from at ages <70 years, where

For peer review only - http://bmjopen.bmj.com/site/about/guidelines.xhtml

For peer review only

men and women with CRD had similar mortality rate. In COVID-19 references without CRD, women had a decreased mortality rate compared to men at all age groups.

Discussion

In this nationwide study including all patients with CRD hospitalised for COVID-19 during all three waves in Sweden there are several important findings.

First, hospitalisation for COVID-19 was associated with a 50% increased risk for mortality both in the CRD and the reference group compared to being hospitalised for influenza in the previous years illustrating that COVID-19 during the first waves was a more severe disease than influenza. The strongest predictor for mortality apart from age was cardiorenal disease (CRD) almost increasing the mortality risk by ~40% in patients with COVID-19. A history of CRD had a similar impact on patients hospitalised with influenza. We also identified peripheral artery disease, previous hospitalisation for pneumonia and diabetes as important risk factors for a fatal outcome after COVID-19.

Secondly, overall female sex compared to male was protective from a fatal outcome following hospitalisation for COVID-19. However in the presence of CRD this gender difference was abolished with an equal mortality rate in ages below 70 years.

This indicates that COVID-19 preventive measures should be prioritised in the elderly but also regardless of sex in those with CRD. Furthermore our data strengthens that national strategies to prevent development of severe CRD could be of importance not only for preventing health care burden from cardiovascular complications but also for reducing complications after severe infectious diseases as COVID-19 and influenza.

For peer review only - http://bmjopen.bmj.com/site/about/guidelines.xhtml

In Sweden, a high-income country with an older population and well-developed access to primary and secondary prevention, heart failure is present in 1-2% and chronic kidney disease in around 4-6%.[14, 15] In the Swedish population with age >70 years heart failure or chronic kidney disease is present in around 10-20%.[16] The CRD population (heart failure and/or CKD diagnosis prior to index) was compared to the reference group burdened with additional comorbidities as coronary artery disease present in 49% and diabetes 34% but when adjusting for other comorbidities CRD was independently associated with mortality.

Furthermore, a vast majority of the patients were treated with RAAS inhibitors and statins and thus considered to be on extensive pharmacological cardiovascular protection.

Hospitalisation for COVID-19 was associated with a significantly higher mortality risk during the first year after hospitalisation than that for hospitalisation for influenza during the previous years, especially during the first two waves where few had access to COVID-19 vaccine. This expands further knowledge from France on COVID-19 by Piroth el al. showing an increased risk of in-hospital mortality compared to that of influenza.[17] The lower mortality rate in the third wave may visualise the effect of COVID-19 vaccination in vulnerable patients and improved experiences on optimal medical strategies. Vaccination is of vast importance to reduce mortality due to influenza. Of note, influenza vaccination in the elderly in Sweden is around 50% where those with CRD and diabetes are prioritised and might have an even higher proportion of vaccination.[18]

In the present cohort CRD was an independent predictor for mortality while in contrast coronary artery disease was not. One explanation for this might be the extensive use of cardiovascular preventive pharmacological therapy. Further, the last decades there has been a more successful prevention of incidence and complications from coronary artery disease,

For peer review only - http://bmjopen.bmj.com/site/about/guidelines.xhtml

while prevention of incidence and complications from CRD has been less effective.[19-22] In addition, compared to an ischaemic event, CRD may have a diffuse disease pattern and therefore being clinically diagnosed later and in a more severe state. Previous treatments for hypertension and coronary artery disease, such as statins and RAAS inhibitors, were associated with a reduced risk for all-cause death in both sexes suggesting that well-controlled risk factors can prevent fatal outcome after COVID-19 infection. Accordingly, one could speculate that if CRD treatment could be improved and CRD being in a less severe state, outcome after a severe infection such as COVID-19 and also influenza would likely be less severe.

Unlike coronary artery disease, history of stroke and PAD were strong risk factors for a worse outcome probably indicating an underlying general severe atherosclerotic burden and frailty. Few studies have analysed the outcome of COVID-19 in patients with heart failure and/or CKD. In accordance with our results, Bergman et al[8] also found that in Sweden high age, diabetes, cardiovascular disease (however not analysing heart failure specifically), hypertension, renal failure and previous pneumonia were associated with all-cause death in hospitalised and non-hospitalised COVID-19 cases although with a shorter median follow-up time than ours (four months).

Interestingly, we found that history of hospitalisation for pneumonia was common among COVID-19 patients, in particular, those with CRD (present in 39%), which was higher than previously reported by Bergman et al[8] (15%). Also, in the influenza population with CRD previous pneumonia was common (47%). After adjusting for age and other risk factors this was also one of the most important predictors for a fatal outcome. This does not seem to be explained by presence of chronic obstructive pulmonary disease (COPD) which was less common (present in around one fifth) and also a less important risk factor. Rather this seems to indicate a patient who is predisposed to a more severe disease development after a

For peer review only - http://bmjopen.bmj.com/site/about/guidelines.xhtml

pulmonary infection. Our data are in line with a recent report from Ballin et al who analysed 30-day mortality in long-term care facility residents in Sweden and also identified previous pneumonia as an important risk factor for mortality in those diagnosed with COVID-19.[23]

Another Swedish study has reported on the selected group admitted to ICU (Svensson et al) and found diabetes, hypertension and obesity as independently associated risk factors for severe COVID-19, when having access to hospital recorded variables in the Swedish Intensive Care Registry (SIR).[7] In the present study, obesity diagnosis (reported in the National Patient Register) was not a predictor for mortality which may reflect "*the obesity paradox"* where overweight in heart failure and in chronic kidney disease may indicate a less catabolic patient.[24] However, this should be interpreted with caution as we have no information about BMI and the reporting of obesity in the National Patient Register is of uncertain representativeness.

In CRD patients hospitalised for COVID-19, almost half of the patients were women. Although significantly older, female sex was protective in survival analysis. However, in the presence of CRD this gender difference was abolished. At ages below 70 years, men and women had similar mortality rates but at ages above 70 men had a higher mortality rate than women. This pattern was not seen in the reference group where women had a lower mortality rate than men at all ages. Thus, in the presence of CRD the protective effect of female sex was not present. Indeed, within women hospitalised for COVID-19 a history of CRD was identified as being the strongest risk factor for a fatal outcome. Presence of coronary artery disease, although less common in women, and previous hospitalisation for pneumonia were other risk factors for a fatal outcome in women with COVID-19.

Strengths and limitations

For peer review only - http://bmjopen.bmj.com/site/about/guidelines.xhtml

The major strength of this study is the use of national registries enabling inclusion of all CRD patients hospitalised for COVID-19 during all three waves in Sweden. Further, we had the ability to compare COVID-19 with characteristics and outcome from previous influenza hospitalisations showing a higher mortality rate from covid-19 when vaccination coverage still was low. Another strength is the comparison with a reference group with similar age and sex structure apart from CRD. A limitation is the inclusion criteria was restricted to hospitalised COVID-19 patients where patients that died with COVID-19 outside hospital care, as in elderly home-care, have been missed. This is also true for the influenza cohort. Further, we have no individual data on influenza vaccination or proportion of patients vaccinated for influenza. Another limitation is the lack of information on laboratory analysis or variables as BMI as these registries do not contain such information. For example, obesity diagnoses are captured only from the National Patient Register and therefore associations between BMI and mortality could not be analysed in this study. However, in real-life situations risk factor from medical history is often the only risk evaluation when prioritising vaccination (as in this report) and seldom based on HbA1c or creatinine levels.

Conclusion

We demonstrate that outcome in patients with COVID-19 and influenza is worse if prior CRD is present independently of age and sex. COVID-19 was associated with higher mortality risk than influenza during the first two waves. Further, the presence of CRD abolished the protective gender effect in women at ages below 70 years. This study highlights the importance of preventing heart failure and chronic kidney disease in the general population to reduce mortality from severe infections in the future.

Author contribution

For peer review only - http://bmjopen.bmj.com/site/about/guidelines.xhtml

The study design was developed by J.B. and M.T. M.T. performed the statistical analyses and managed the database. V.R. and A.N. finalised the manuscript after adjustments by J.B., M.T., J.W.E., R.K., T.N. and D.N. All authors made a substantial contribution to this article and was involved in the interpretation of the results.

Declaration of interests

The authors declared the following potential conflicts of interest with respect to the research, authorship and publication of this article: V.R. has received honoraria on expert group participation from Astra Zeneca, Novo Nordisk and Boehringer Ingelheim. A.N. has received honoraria from Astra Zeneca, Merck Sharp & Dohme, Eli Lilly and Company, Novo Nordisk and Boehringer Ingelheim on expert group participation. T.N. has received unrestricted grants from AstraZeneca and NovoNordisk and has served on national advisory boards of Abbot, Amgen, Novo Nordisk, Sanofi-Aventis, Eli Lilly, MSD and Boehringer Ingelheim. J.W.E. has received research support or honoraria from AstraZeneca, NovoNordisk, Bayer, Ilya Pharma, Merck-Sharp & Dohme, Boehringer-Ingelheim. R.K. and D.N. reports no conflicts of interest. J.B. holds a full-time position at AstraZeneca as an epidemiologist. M.T. holds a full-time position by an independent statistical consultant company, Statisticon AB, Uppsala, Sweden, of which AstraZeneca Nordic is a client.

For peer review only

Funding

The study was fully sponsored by AstraZeneca. V.R. has received funding from the Department of Research and Development Region Kronoberg and the Family Kamprad Foundation. A.N. has received funding from the Family Kamprad Foundation and from Capio S:t Görans hospital.

For peer review only - http://bmjopen.bmj.com/site/about/guidelines.xhtml

Acknowledgements

The authors are grateful to Susanna Jerström and Helena Goike at AstraZeneca for logistic support and valuable comments on the manuscript. Urban Olsson, Statisticon AB, is acknowledged for database management.

Ethics Approval Statement

The study was approved by the Stockholm Regional Ethics Committee (reference numbers 2020-05714 and 2013/2206-31). Ethical permission approved a reference group to a collected population with cardiorenal disease (CRD; heart failure and/or chronic kidney disease [CARE-19]) but not a reference group to COVID-19 or influenza patients.

Data availability statement

All data relevant to the study are included in the article or uploaded as supplementary information.

For peer review only

References

1. Phelan AL, Katz R, Gostin LO. The Novel Coronavirus Originating in Wuhan, China: Challenges for Global Health Governance. *JAMA* 2020;323(8):709-10.

2. Guan WJ, Ni ZY, Hu Y, Liang WH, Ou CQ, He JX, Liu L, Shan H, Lei CL, Hui DSC, Du B, Li LJ, Zeng G, Yuen KY, Chen RC, Tang CL, Wang T, Chen PY, Xiang J, Li SY, Wang JL, Liang ZJ, Peng YX, Wei L, Liu Y, Hu YH, Peng P, Wang JM, Liu JY, Chen Z, Li G, Zheng ZJ, Qiu SQ, Luo J, Ye CJ, Zhu SY, Zhong NS; China Medical Treatment Expert Group for Covid-19. Clinical Characteristics of Coronavirus Disease 2019 in China. *N Engl J Med* 2020;382(18):1708-20.

3. Grasselli G, Greco M, Zanella A, Albano G, Antonelli M, Bellani G, Bonanomi E, Cabrini L, Carlesso E, Castelli G, Cattaneo S, Cereda D, Colombo S, Coluccello A, Crescini G, Forastieri Molinari A, Foti G, Fumagalli R, Iotti GA, Langer T, Latronico N, Lorini FL, Mojoli F, Natalini G, Pessina CM, Ranieri VM, Rech R, Scudeller L, Rosano A, Storti E, Thompson BT, Tirani M, Villani PG, Pesenti A, Cecconi M; COVID-19 Lombardy ICU Network. Risk Factors Associated With Mortality Among Patients With COVID-19 in Intensive Care Units in Lombardy, Italy. *JAMA Intern Med* 2020;180(10):1345-55.

4. Evans D, Inglesby P, Cockburn J, McDonald HI, MacKenna B, Tomlinson L, Douglas IJ, Rentsch CT, Mathur R, Wong AYS, Grieve R, Harrison D, Forbes H, Schultze A, Croker R, Parry J, Hester F, Harper S, Perera R, Evans SJW, Smeeth L, Goldacre B. Factors associated with COVID-19-related death using OpenSAFELY. *Nature* 2020;584(7821):430-6.

5. Reilev M, Kristensen KB, Pottegård A, Lund LC, Hallas J, Ernst MT, Christiansen CF, Sørensen HT, Johansen NB, Brun NC, Voldstedlund M, Støvring H, Thomsen MK, Christensen S, Gubbels S, Krause TG, Mølbak K, Thomsen RW. Characteristics and

For peer review only

For peer review only - http://bmjopen.bmj.com/site/about/guidelines.xhtml

predictors of hospitalization and death in the first 11 122 cases with a positive RT-PCR test for SARS-CoV-2 in Denmark: a nationwide cohort. *Int J Epidemiol* 2020;49(5):1468-81.

6. Cummings MJ, Baldwin MR, Abrams D, Jacobson SD, Meyer BJ, Balough EM, Aaron JG, Claassen J, Rabbani LE, Hastie J, Hochman BR, Salazar-Schicchi J, Yip NH, Brodie D, O'Donnell MR. Epidemiology, clinical course, and outcomes of critically ill adults with COVID-19 in New York City: a prospective cohort study. *Lancet* 2020;395(10239):1763-70.

7. Svensson P, Hofmann R, Häbel H, Jernberg T, Nordberg P. Association between cardiometabolic disease and severe COVID-19: a nationwide case-control study of patients requiring invasive mechanical ventilation. *BMJ Open* 2021;11(2):e044486.

8. Bergman J, Ballin M, Nordström A, Nordström P. Risk factors for COVID-19 diagnosis, hospitalization, and subsequent all-cause mortality in Sweden: a nationwide study. *Eur J Epidemiol* 2021;36(3):287-98.

9. Al-Salameh A, Lanoix JP, Bennis Y, Andrejak C, Brochot E, Deschasse G, Dupont H, Goeb V, Jaureguy M, Lion S, Maizel J, Moyet J, Vaysse B, Desailloud R, Ganry O, Schmit JL, Lalau JD. Characteristics and outcomes of COVID-19 in hospitalized patients with and without diabetes. *Diabetes Metab Res Rev* 2021;37(3):e3388.

10. Rawshani A, Kjölhede EA, Rawshani A, Sattar N, Eeg-Olofsson K, Adiels M, Ludvigsson J, Lindh M, Gisslén M, Hagberg E, Lappas G, Eliasson B, Rosengren A. Severe COVID-19 in people with type 1 and type 2 diabetes in Sweden: A nationwide retrospective cohort study. *Lancet Reg Health Eur* 2021;4:100105.

11. Barron E, Bakhai C, Kar P, Weaver A, Bradley D, Ismail H, Knighton P, Holman N, Khunti K, Sattar N, Wareham NJ, Young B, Valabhji J. Associations of type 1 and type 2 diabetes with COVID-19-related mortality in England: a whole-population study. *Lancet Diabetes Endocrinol* 2020;8(10):813-22.

For peer review only

For peer review only - http://bmjopen.bmj.com/site/about/guidelines.xhtml

12. Holman N, Knighton P, Kar P, O'Keefe J, Curley M, Weaver A, Barron E, Bakhai C, Khunti K, Wareham NJ, Sattar N, Young B, Valabhji J. Risk factors for COVID-19-related mortality in people with type 1 and type 2 diabetes in England: a population-based cohort study. *Lancet Diabetes Endocrinol* 2020;8(10):823-33.

13. Rangaswami J, Bhalla V, Blair JEA, Chang TI, Costa S, Lentine KL, Lerma EV, Mezue K, Molitch M, Mullens W, Ronco C, Tang WHW, McCullough PA; American Heart Association Council on the Kidney in Cardiovascular Disease and Council on Clinical Cardiology. Cardiorenal Syndrome: Classification, Pathophysiology, Diagnosis, and Treatment Strategies: A Scientific Statement From the American Heart Association. *Circulation* 2019;139(16):e840-e878.

14. Seferović PM, Vardas P, Jankowska EA, Maggioni AP, Timmis A, Milinković I, Polovina M, Gale CP, Lund LH, Lopatin Y, Lainscak M, Savarese G, Huculeci R, Kazakiewicz D, Coats AJS; National Heart Failure Societies of the ESC member countries (see Appendix). The Heart Failure Association Atlas: Heart Failure Epidemiology and Management Statistics 2019. *Eur J Heart Fail* 2021;23(6):906-14.

15. Zarrinkoub R, Wettermark B, Wändell P, Mejhert M, Szulkin R, Ljunggren G, Kahan T. The epidemiology of heart failure, based on data for 2.1 million inhabitants in Sweden. *Eur J Heart Fail* 2013;15(9):995-1002.

16. Gasparini A, Evans M, Coresh J, Grams ME, Norin O, Qureshi AR, Runesson B, Barany P, Ärnlöv J, Jernberg T, Wettermark B, Elinder CG, Carrero JJ. Prevalence and recognition of chronic kidney disease in Stockholm healthcare. *Nephrol Dial Transplant* 2016;31(12):2086-94.

17. Piroth L, Cottenet J, Mariet AS, Bonniaud P, Blot M, Tubert-Bitter P, Quantin C. Comparison of the characteristics, morbidity, and mortality of COVID-19 and seasonal

For peer review only

For peer review only - http://bmjopen.bmj.com/site/about/guidelines.xhtml

influenza: a nationwide, population-based retrospective cohort study. *Lancet Respir Med* 2021;9(3):251-9.

18. The Public Health Agency of Sweden. Available from: https://www.folkhalsomyndigheten.se/folkhalsorapportering-statistik/statistikdatabaser-och-visualisering/vaccinationsstatistik/statistik-for-influensavaccinationer/. Date of access 10 November, 2021.

19. Yang D, Dzayee DA, Beiki O, de Faire U, Alfredsson L, Moradi T. Incidence and case fatality after day 28 of first time myocardial infarction in Sweden 1987-2008. *Eur J Prev Cardiol* 2012;19(6):1304-15.

20. Szummer K, Wallentin L, Lindhagen L, Alfredsson J, Erlinge D, Held C, James S, Kellerth T, Lindahl B, Ravn-Fischer A, Rydberg E, Yndigegn T, Jernberg T. Improved outcomes in patients with ST-elevation myocardial infarction during the last 20 years are related to implementation of evidence-based treatments: experiences from the SWEDEHEART registry 1995-2014. *Eur Heart J* 2017;38(41):3056-65.

21. Szummer K, Wallentin L, Lindhagen L, Alfredsson J, Erlinge D, Held C, James S, Kellerth T, Lindahl B, Ravn-Fischer A, Rydberg E, Yndigegn T, Jernberg T. Relations between implementation of new treatments and improved outcomes in patients with non-ST-elevation myocardial infarction during the last 20 years: experiences from SWEDEHEART registry 1995 to 2014. *Eur Heart J* 2018;39(42):3766-76.

22. House AA, Wanner C, Sarnak MJ, Piña IL, McIntyre CW, Komenda P, Kasiske BL, Deswal A, deFilippi CR, Cleland JGF, Anker SD, Herzog CA, Cheung M, Wheeler DC, Winkelmayer WC, McCullough PA; Conference Participants. Heart failure in chronic kidney disease: conclusions from a Kidney Disease: Improving Global Outcomes (KDIGO) Controversies Conference. *Kidney Int* 2019;95(6):1304-17.

For peer review only

For peer review only - http://bmjopen.bmj.com/site/about/guidelines.xhtml

23. Ballin M, Bergman J, Kivipelto M, Nordström A, Nordström P. Excess Mortality After COVID-19 in Swedish Long-Term Care Facilities. *J Am Med Dir Assoc* 2021;22(8):1574-80.

24. Ritsinger V, Brismar K, Malmberg K, Mellbin L, Näsman P, Rydén L, Söderberg S, Tenerz Å, Norhammar A. Elevated levels of adipokines predict outcome after acute myocardial infarction - a long-term follow up of the GAMI cohort. *Diab Vasc Dis Res* 2017;14(2):77-87.

For peer review only

For peer review only - http://bmjopen.bmj.com/site/about/guidelines.xhtml

Figure legends

Figure 1. Cumulative incidence of all-cause death in COVID-19 patients between the years 2020-2021 with cardiorenal disease (CRD; blue) and without CRD (dotted blue) and in influenza patients between the years 2015-2019 with CRD (green) and without CRD (dotted green).

Figure 2. All-cause death in patients with cardiorenal disease and COVID-19 or influenza by the three COVID-19 waves in Sweden.

Figure 3A. Cumulative incidence of all-cause death in men with COVID-19 between the years 2020-2021 with cardiorenal disease (CRD; blue) and without CRD (dotted blue) and in men with influenza between the years 2015-2019 with CRD (green) and without CRD (dotted green).

Figure 3B. Cumulative incidence of all-cause death in women with COVID-19 between the years 2020-2021 with cardiorenal disease (CRD; blue) and without CRD (dotted blue) and in women with influenza between the years 2015-2019 with CRD (green) and without CRD (dotted green).

Figure 4. Adjusted associated HR (95% CI) for all-cause death in men and women and mortality associated with COVID-19 vs. Influenza. .

Figure 5. All-cause death rate by sex and age groups stratified by cardiorenal disease (CRD) in patients with COVID-19.

For peer review only - http://bmjopen.bmj.com/site/about/guidelines.xhtml

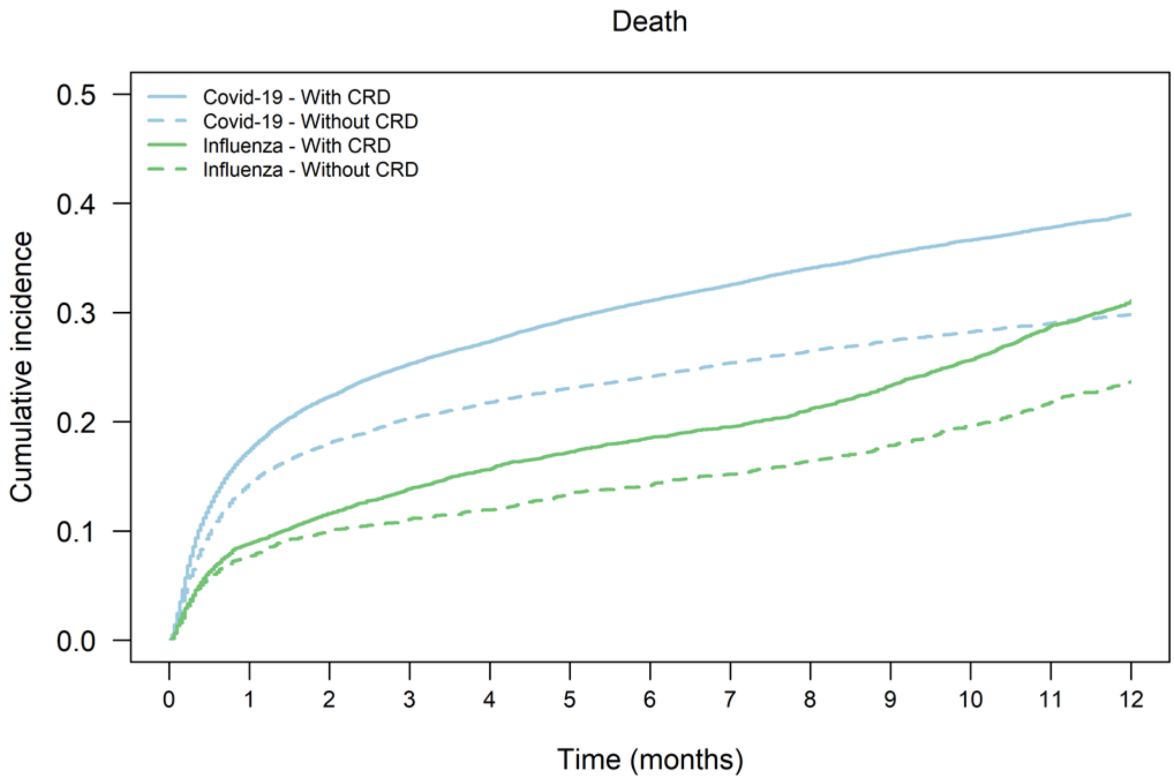
Death
Covid-19 - With CRD
Covid-19 - Without CRD
Influenza - With CRD
Influenza - Without CRD
Cumulative incidence
0.5
0.4
0.3
0.2
0.1
0.0
0 1 2 3 4 5 6 7 8 9 10 11 12
Time (months)

review only

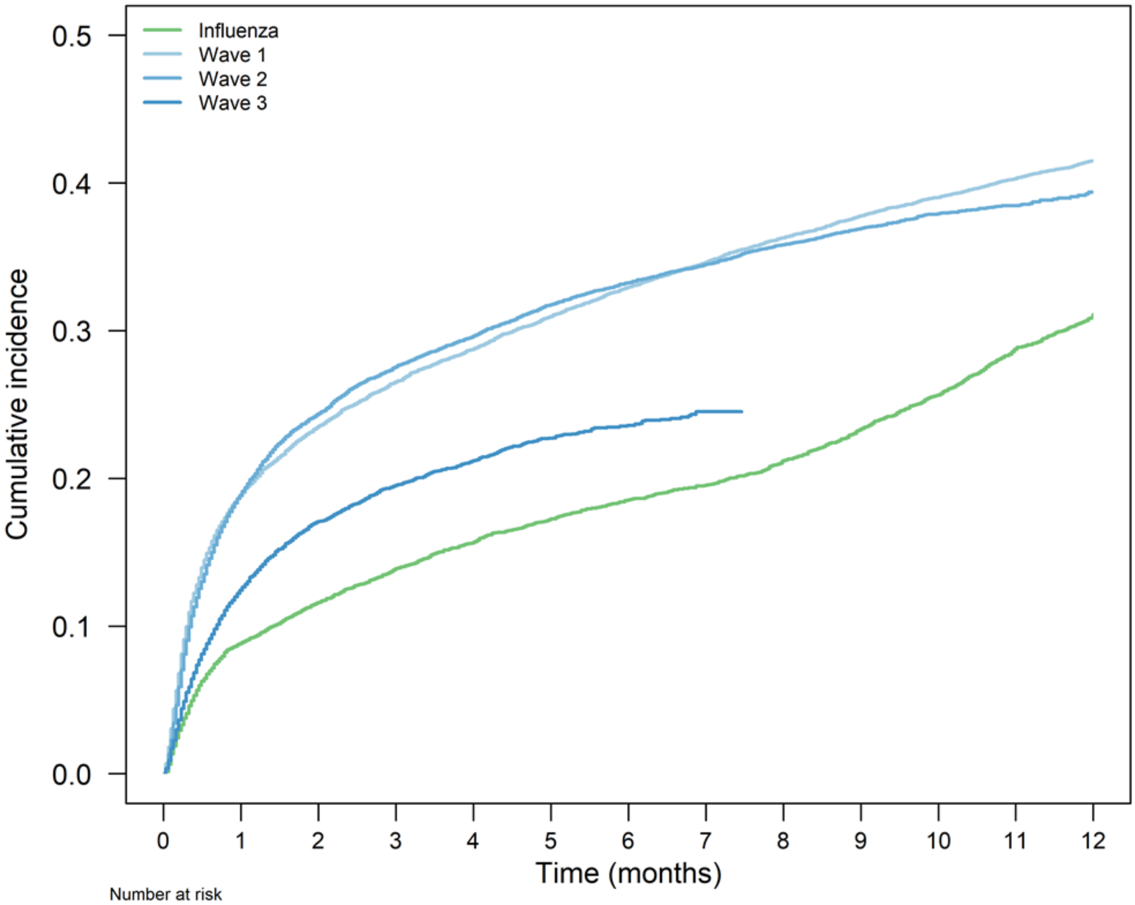

Number at risk

	0	2	4	6	8	10	12
Influenza	5983	5243	5003	4829	4655	4175	3529
Wave 1	10918	8360	7783	7324	6957	6657	6386
Wave 2	11422	8646	8045	7626	6546	2596	439
Wave 3	6855	5583	3990	1704			

A.

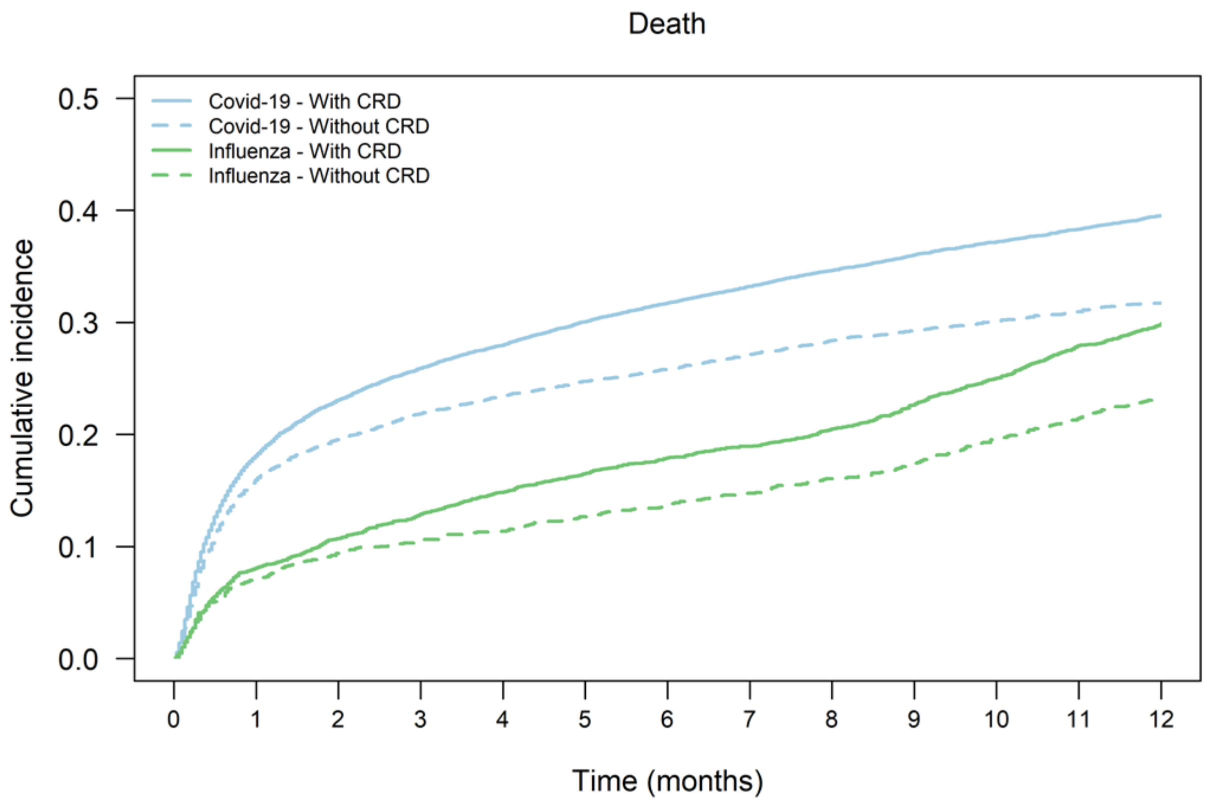
Death
Covid-19 - With CRD
Covid-19 - Without CRD
Influenza - With CRD
Influenza - Without CRD
Cumulative incidence
0.0
0.1
0.2
0.3
0.4
0.5
0 1 2 3 4 5 6 7 8 9 10 11 12
Time (months)

B.

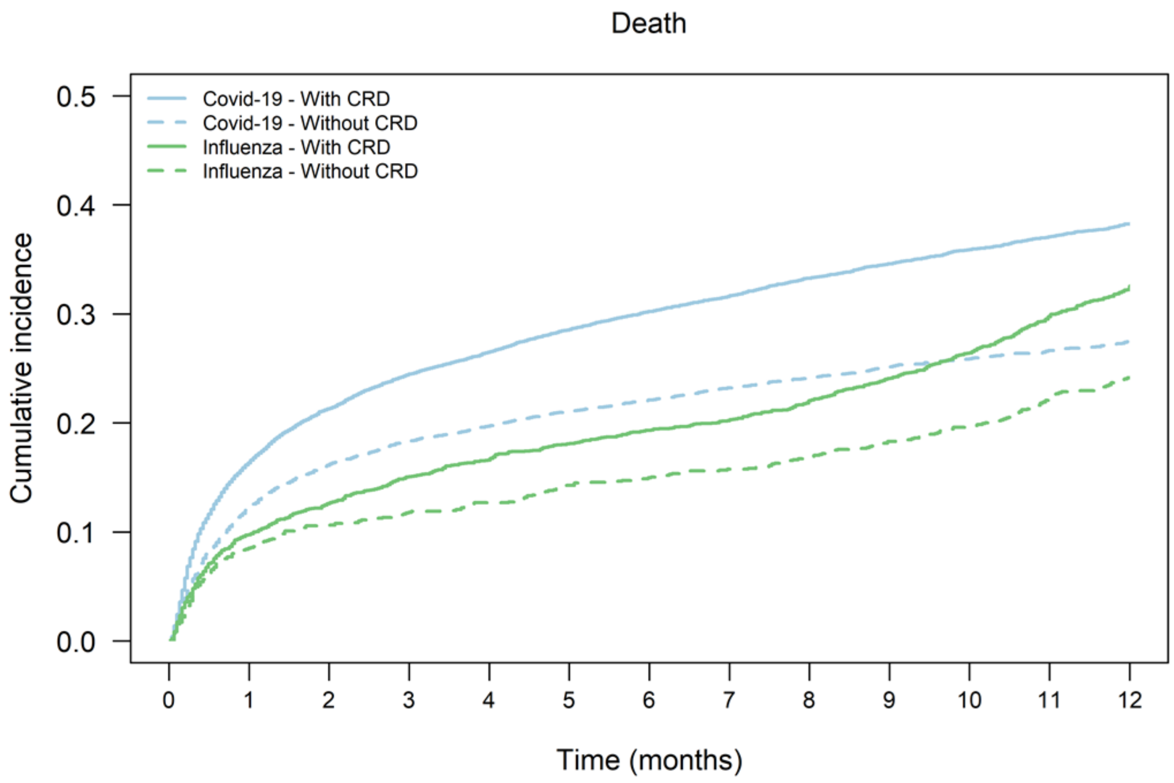
Death
Covid-19 - With CRD
Covid-19 - Without CRD
Influenza - With CRD
Influenza - Without CRD
Cumulative incidence
0.0
0.1
0.2
0.3
0.4
0.5
0 1 2 3 4 5 6 7 8 9 10 11 12
Time (months)

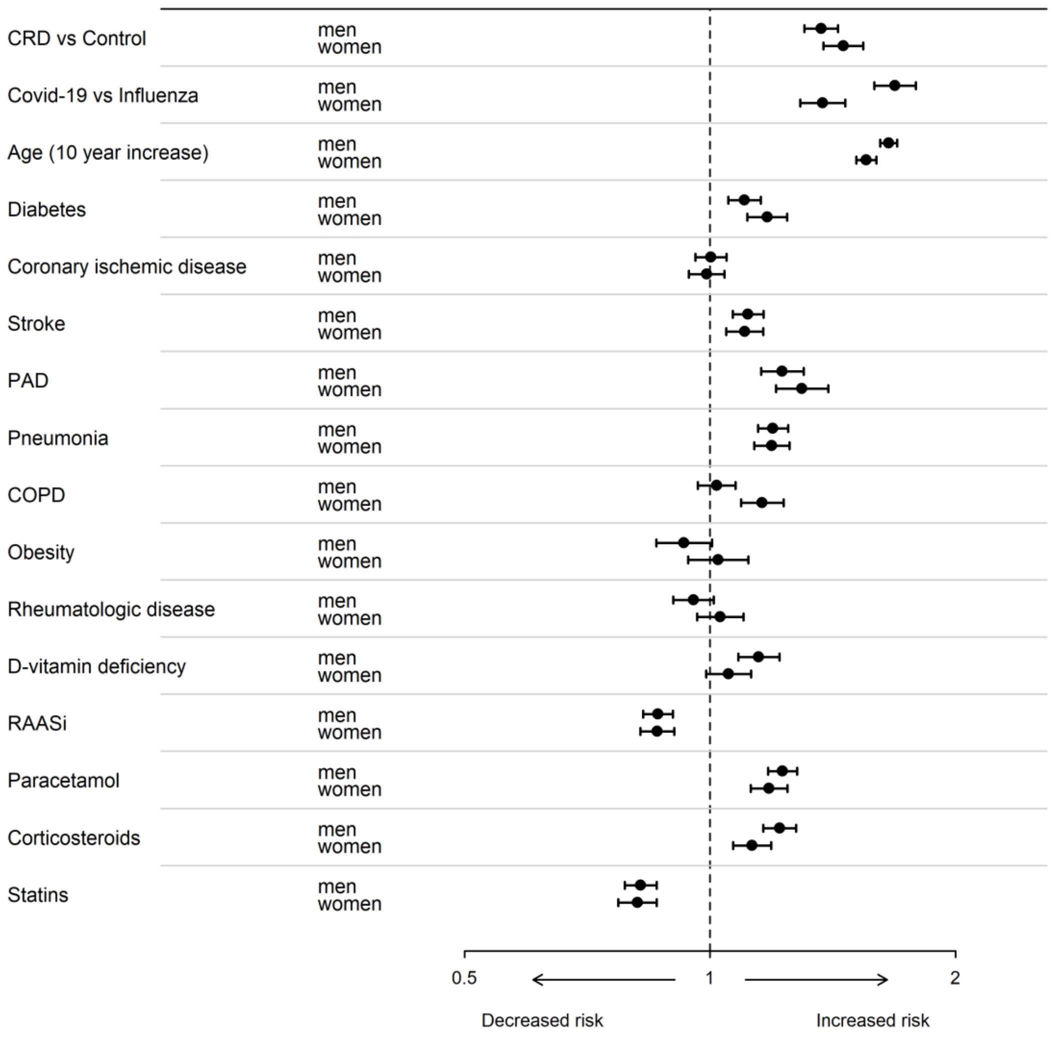
CRD vs Control
Covid-19 vs Influenza
Age (10 year increase)
Diabetes
Coronary ischemic disease
Stroke
PAD
Pneumonia
COPD
Obesity
Rheumatologic disease
D-vitamin deficiency
RAASi
Paracetamol
Corticosteroids
Statins
men
women
0.5
1
2
Decreased risk
Increased risk

only

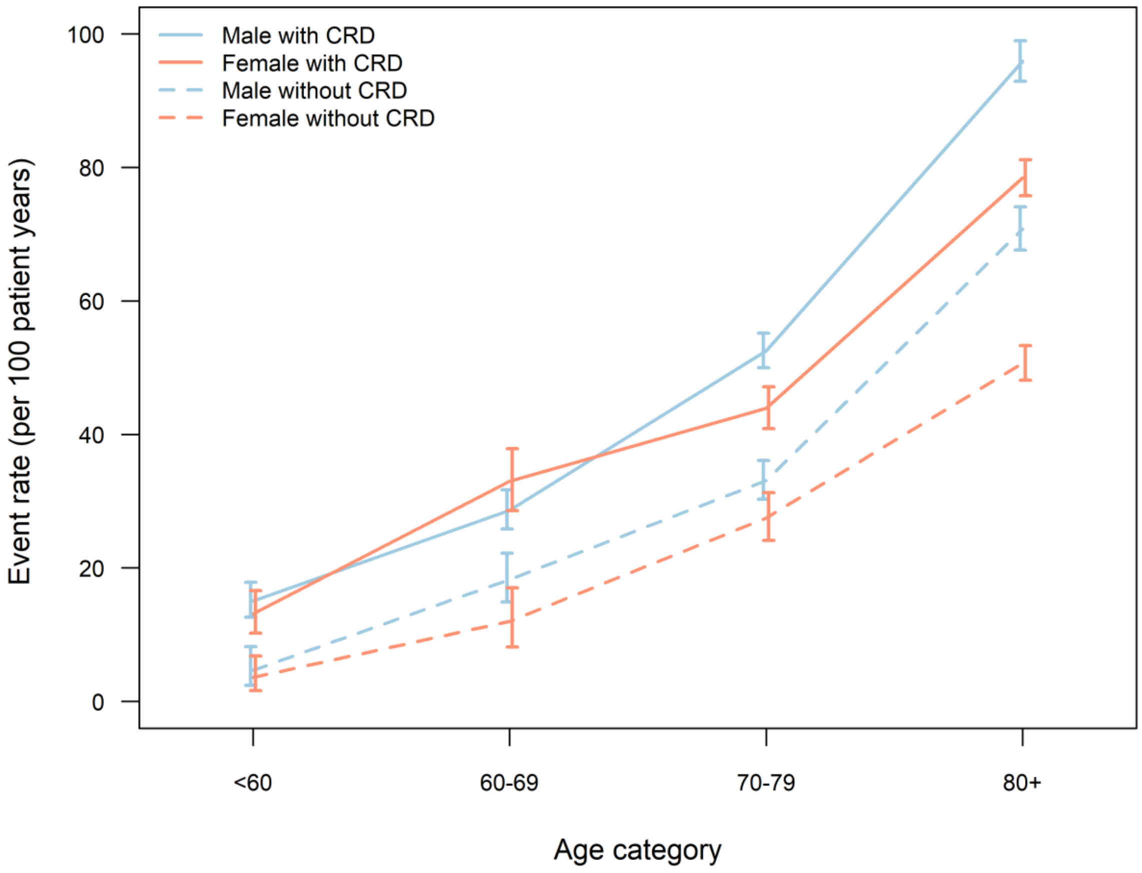

Male with CRD
Female with CRD
Male without CRD
Female without CRD
Event rate (per 100 patient years)
0
20
40
60
80
100
<60
60-69
70-79
80+
Age category

Supplemental material

Table 1. Adjusted HR and 95% CI for all-cause death in COVID-19 patients.

	Hazard ratio	95% CI	P-value
Cardiorenal disease (CRD) vs. no CRD	1.41	(1.36-1.46)	<0.001
Age per 10 years increase	1.61	(1.58-1.64)	<0.001
Female sex	0.76	(0.73-0.78)	<0.001
Diabetes	1.13	(1.09-1.17)	<0.001
Coronary artery disease	1.00	(0.97-1.03)	0.881
Stroke	1.11	(1.07-1.15)	<0.001
Peripheral artery disease (PAD)	1.25	(1.19-1.31)	<0.001
Pneumonia	1.19	(1.16-1.23)	<0.001
Chronic obstructive pulmonary disease (COPD)	1.08	(1.04-1.13)	<0.001
Obesity	0.97	(0.92-1.03)	0.335
Rheumatologic disease	0.99	(0.95-1.03)	0.570
D-vitamin deficiency	1.10	(1.06-1.15)	<0.001
RAAS inhibitors	0.86	(0.83-0.89)	<0.001
Paracetamol	1.21	(1.17-1.25)	<0.001
Corticosteroids	1.18	(1.14-1.22)	<0.001
Statins	0.82	(0.79-0.85)	<0.001

For peer review only

For peer review only - http://bmjopen.bmj.com/site/about/guidelines.xhtml

Table 2. Baseline characteristics in men with COVID-19 or influenza with and without cardiorenal disease (CRD).

	Covid-19 with CRD n=16721	Covid-19 without CRD n=8670	Influenza with CRD n=3283	Influenza without CRD n=1638
Age, years, mean (SD)	77.7 (11.7)	80.1 (10.4)	78.1 (11.9)	82.1 (9.1)
Age, years, median (min-max)	79 (18-103)	82 (20-103)	80 (20-106)	83 (24-104)
Females, n (%)	0 (0)	0 (0)	0 (0)	0 (0)
Year				
2020, n (%)	11329 (68)	5477 (63)	0 (0)	0 (0)
2021, n (%)	5392 (32)	3193 (37)	0 (0)	0 (0)
2015, n (%)	0 (0)	0 (0)	515 (16)	246 (15)
2016, n (%)	0 (0)	0 (0)	459 (14)	227 (14)
2017, n (%)	0 (0)	0 (0)	624 (19)	328 (20)
2018, n (%)	0 (0)	0 (0)	1051 (32)	531 (32)
2019, n (%)	0 (0)	0 (0)	634 (19)	306 (19)
In-hospital stay, days, median (IQR)	6 (4-11)	6 (4-10)	6 (4-10)	5 (3-9)
Heart failure, n (%)	13275 (79)	0 (0)	2648 (81)	0 (0)
Chronic kidney disease (CKD), n (%)	7025 (42)	0 (0)	1260 (38)	0 (0)
Dialysis, n (%)	1174 (7)	0 (0)	350 (11)	0 (0)
Coronary artery disease, n (%)	8810 (53)	2270 (26)	1882 (57)	485 (30)
Stroke, n (%)	4747 (28)	2141 (25)	967 (29)	441 (27)
Atrial fibrillation, n (%)	9102 (54)	1904 (22)	1759 (54)	379 (23)
Peripheral artery disease (PAD), n (%)	2127 (13)	488 (6)	422 (13)	86 (5)
Diabetes, n (%)	6158 (37)	1863 (21)	1104 (34)	372 (23)
Chronic obstructive pulmonary disease (COPD), n (%)	3243 (19)	772 (9)	688 (21)	161 (10)
Any history of pneumonia (viral/bacterial), n (%)	6276 (38)	1681 (19)	1519 (46)	475 (29)
Any history of thromboembolism (DVT or PE), n (%)	2120 (13)	741 (9)	399 (12)	139 (8)
Vitamin-D deficiency, n (%)	3081 (18)	450 (5)	610 (19)	70 (4)
Cancer, n (%)	6372 (38)	3300 (38)	1175 (36)	581 (35)
Any of ACEi/ARB/MRA/ARNi/SGLT2i/β-blocker, n (%)*	15056 (90)	5322 (61)	2974 (91)	1023 (62)
RAAS inhibitor, n (%)	11293 (68)	4027 (46)	2307 (70)	729 (45)
Statins, n (%)	9733 (58)	3521 (41)	1875 (57)	655 (40)
Corticosteroids, n (%)	4585 (27)	1492 (17)	1081 (33)	339 (21)
Paracetamol, n (%)	8850 (53)	3497 (40)	1667 (51)	656 (40)
Modulating antineoplastic and hormone drugs, n (%)	1182 (7)	706 (8)	210 (6)	110 (7)

* Any of Angiotensin-converting enzyme (ACE) inhibitors, Angiotensin II receptor blockers (ARBs), Mineralocorticoid receptor antagonists (MRA), Angiotensin receptor-neprilysin inhibitors (ARNi), Sodium-glucose Cotransporter-2 (SGLT2) inhibitors and Beta blockers.

only

For peer review only - http://bmjopen.bmj.com/site/about/guidelines.xhtml

Table 3. Baseline characteristics in women with COVID-19 or influenza with and without cardiorenal disease (CRD).

	Covid-19 with CRD n=12474	Covid-19 without CRD n=7001	Influenza with CRD n=2700	Influenza without CRD n=1276
Age, years, mean (SD)	80.9 (12.0)	82.6 (12.3)	81.1 (12.4)	84.3 (9.6)
Age, years, median (min-max)	83 (18-107)	85 (18-106)	84 (20-102)	86 (32-107)
Females, n (%)	12474 (100)	7001 (100)	2700 (100)	1276 (100)
Year				
2020, n (%)	8400 (67)	4437 (63)	0 (0)	0 (0)
2021, n (%)	4074 (33)	2564 (37)	0 (0)	0 (0)
2015, n (%)	0 (0)	0 (0)	431 (16)	197 (15)
2016, n (%)	0 (0)	0 (0)	402 (15)	147 (12)
2017, n (%)	0 (0)	0 (0)	530 (20)	276 (22)
2018, n (%)	0 (0)	0 (0)	839 (31)	432 (34)
2019, n (%)	0 (0)	0 (0)	498 (18)	224 (18)
In-hospital stay, days, median (IQR)	7 (4-11)	6 (4-10)	7 (4-11)	6 (4-11)
Heart failure, n (%)	10374 (83)	0 (0)	2323 (86)	0 (0)
Chronic kidney disease (CKD), n (%)	4288 (34)	0 (0)	766 (28)	0 (0)
Dialysis, n (%)	603 (5)	0 (0)	170 (6)	0 (0)
Coronary artery disease, n (%)	5504 (44)	1335 (19)	1311 (49)	301 (24)
Stroke, n (%)	3402 (27)	1514 (22)	724 (27)	291 (23)
Atrial fibrillation, n (%)	6415 (51)	1274 (18)	1418 (53)	266 (21)
Peripheral artery disease (PAD), n (%)	1440 (12)	331 (5)	259 (10)	72 (6)
Diabetes, n (%)	3892 (31)	1111 (16)	739 (27)	208 (16)
Chronic obstructive pulmonary disease (COPD), n (%)	2839 (23)	649 (9)	649 (24)	163 (13)
Any history of pneumonia (viral/bacterial), n (%)	4799 (38)	1256 (18)	1270 (47)	380 (30)
Any history of thromboembolism (DVT or PE), n (%)	1824 (15)	666 (10)	393 (15)	131 (10)
Vitamin-D deficiency, n (%)	2699 (22)	664 (9)	474 (18)	86 (7)
Cancer, n (%)	4577 (37)	2480 (35)	906 (34)	433 (34)
Any of ACEi/ARB/MRA/ARNi/SGLT2i/β-blocker, n (%)*	11131 (89)	4355 (62)	2427 (90)	840 (66)
RAAS inhibitor, n (%)	7992 (64)	3095 (44)	1766 (65)	570 (45)
Statins, n (%)	5437 (44)	2045 (29)	1059 (39)	373 (29)
Corticosteroids, n (%)	3927 (31)	1418 (20)	1015 (38)	360 (28)
Paracetamol, n (%)	8466 (68)	3828 (55)	1828 (68)	728 (57)
Modulating antineoplastic and hormone drugs, n (%)	516 (4)	256 (4)	89 (3)	35 (3)

* Any of Angiotensin-converting enzyme (ACE) inhibitors, Angiotensin II receptor blockers (ARBs), Mineralocorticoid receptor antagonists (MRA), Angiotensin receptor-neprilysin inhibitors (ARNi), Sodium-glucose Cotransporter-2 (SGLT2) inhibitors and Beta blockers.

For peer review only - http://bmjopen.bmj.com/site/about/guidelines.xhtml

STROBE Statement—checklist of items that should be included in reports of observational studies

	Item No	Recommendation	Page No
**Title and abstract**	1	(*a*) Indicate the study's design with a commonly used term in the title or the abstract	1
		(*b*) Provide in the abstract an informative and balanced summary of what was done and what was found	1
**Introduction**			
Background/rationale	2	Explain the scientific background and rationale for the investigation being reported	1
Objectives	3	State specific objectives, including any prespecified hypotheses	4-5
**Methods**			
Study design	4	Present key elements of study design early in the paper	5-6
Setting	5	Describe the setting, locations, and relevant dates, including periods of recruitment, exposure, follow-up, and data collection	5-7
Participants	6	(*a*) *Cohort study*—Give the eligibility criteria, and the sources and methods of selection of participants. Describe methods of follow-up *Case-control study*—Give the eligibility criteria, and the sources and methods of case ascertainment and control selection. Give the rationale for the choice of cases and controls *Cross-sectional study*—Give the eligibility criteria, and the sources and methods of selection of participants	5-7
		(*b*) *Cohort study*—For matched studies, give matching criteria and number of exposed and unexposed *Case-control study*—For matched studies, give matching criteria and the number of controls per case	5-7
Variables	7	Clearly define all outcomes, exposures, predictors, potential confounders, and effect modifiers. Give diagnostic criteria, if applicable	5-7
Data sources/ measurement	8*	For each variable of interest, give sources of data and details of methods of assessment (measurement). Describe comparability of assessment methods if there is more than one group	*5-7*
Bias	9	Describe any efforts to address potential sources of bias	16
Study size	10	Explain how the study size was arrived at	5-6
Quantitative variables	11	Explain how quantitative variables were handled in the analyses. If applicable, describe which groupings were chosen and why	7-8
Statistical methods	12	(*a*) Describe all statistical methods, including those used to control for confounding	7-8
		(*b*) Describe any methods used to examine subgroups and interactions	7-8
		(*c*) Explain how missing data were addressed	7-8
		(*d*) *Cohort study*—If applicable, explain how loss to follow-up was addressed *Case-control study*—If applicable, explain how matching of cases and controls was addressed *Cross-sectional study*—If applicable, describe analytical methods taking account of sampling strategy	7-8
		(*e*) Describe any sensitivity analyses	7-8

Continued on next page

For peer review only - http://bmjopen.bmj.com/site/about/guidelines.xhtml

**Results**			
Participants	13*	(a) Report numbers of individuals at each stage of study—eg numbers potentially eligible, examined for eligibility, confirmed eligible, included in the study, completing follow-up, and analysed	5-7
		(b) Give reasons for non-participation at each stage	5-7
		(c) Consider use of a flow diagram	
Descriptive data	14*	(a) Give characteristics of study participants (eg demographic, clinical, social) and information on exposures and potential confounders	8-9
		(b) Indicate number of participants with missing data for each variable of interest	8-9
		(c) *Cohort study*—Summarise follow-up time (eg, average and total amount)	8-9
Outcome data	15*	*Cohort study*—Report numbers of outcome events or summary measures over time	
		Case-control study—Report numbers in each exposure category, or summary measures of exposure	*8-9*
		Cross-sectional study—Report numbers of outcome events or summary measures	
Main results	16	(*a*) Give unadjusted estimates and, if applicable, confounder-adjusted estimates and their precision (eg, 95% confidence interval). Make clear which confounders were adjusted for and why they were included	10-11
		(*b*) Report category boundaries when continuous variables were categorized	10-11
		(*c*) If relevant, consider translating estimates of relative risk into absolute risk for a meaningful time period	10-11
Other analyses	17	Report other analyses done—eg analyses of subgroups and interactions, and sensitivity analyses	10-11
**Discussion**			
Key results	18	Summarise key results with reference to study objectives	12-15
Limitations	19	Discuss limitations of the study, taking into account sources of potential bias or imprecision. Discuss both direction and magnitude of any potential bias	12-15
Interpretation	20	Give a cautious overall interpretation of results considering objectives, limitations, multiplicity of analyses, results from similar studies, and other relevant evidence	12-15
Generalisability	21	Discuss the generalisability (external validity) of the study results	12-15
**Other information**			
Funding	22	Give the source of funding and the role of the funders for the present study and, if applicable, for the original study on which the present article is based	17

*Give information separately for cases and controls in case-control studies and, if applicable, for exposed and unexposed groups in cohort and cross-sectional studies.

Note: An Explanation and Elaboration article discusses each checklist item and gives methodological background and published examples of transparent reporting. The STROBE checklist is best used in conjunction with this article (freely available on the Web sites of PLoS Medicine at http://www.plosmedicine.org/, Annals of Internal Medicine at http://www.annals.org/, and Epidemiology at http://www.epidem.com/). Information on the STROBE Initiative is available at www.strobe-statement.org.

For peer review only - http://bmjopen.bmj.com/site/about/guidelines.xhtml

BMJ Open

History of heart failure and chronic kidney disease and risk of all-cause death after COVID-19 during the first three waves of the pandemic in comparison with influenza outbreaks in Sweden: a registry-based, retrospective, case-control study

Journal:	*BMJ Open*
Manuscript ID	bmjopen-2022-069037.R1
Article Type:	Original research
Date Submitted by the Author:	14-Feb-2023
Complete List of Authors:	Ritsinger, Viveca; Karolinska Institutet Institutionen for medicin Solna, Cardiology Unit; Region Kronoberg, Department of Research and Development<br>Bodegård, Johan; AstraZeneca PLC<br>Kristofi, Robin; Uppsala University, Department of Medical Sciences, Clinical Diabetes and Metabolism<br>Thuresson, Marcus; Uppsala universitet Statistiska institutionen, Department of Statistics<br>Nathanson, David; Department of Medicine, Huddinge, Karolinska Institute<br>Nyström, Thomas; Södersjukhuset, Department of Clinical Science and Education<br>Eriksson, Jan; Uppsala University, Department of Medical Sciences, Clinical Diabetes and Metabolism<br>Norhammar, Anna; Karolinska Institutet Institutionen for medicin Solna, Cardiology Unit; Capio Saint Göran's Hospital
**Primary Subject Heading**:	Cardiovascular medicine
Secondary Subject Heading:	Infectious diseases, Epidemiology, Renal medicine
Keywords:	COVID-19, Heart failure < CARDIOLOGY, EPIDEMIOLOGY, Chronic renal failure < NEPHROLOGY

SCHOLARONE™
Manuscripts

I, the Submitting Author has the right to grant and does grant on behalf of all authors of the Work (as defined in the below author licence), an exclusive licence and/or a non-exclusive licence for contributions from authors who are: i) UK Crown employees; ii) where BMJ has agreed a CC-BY licence shall apply, and/or iii) in accordance with the terms applicable for US Federal Government officers or employees acting as part of their official duties; on a worldwide, perpetual, irrevocable, royalty-free basis to BMJ Publishing Group Ltd ("BMJ") its licensees and where the relevant Journal is co-owned by BMJ to the co-owners of the Journal, to publish the Work in this journal and any other BMJ products and to exploit all rights, as set out in our licence.

The Submitting Author accepts and understands that any supply made under these terms is made by BMJ to the Submitting Author unless you are acting as an employee on behalf of your employer or a postgraduate student of an affiliated institution which is paying any applicable article publishing charge ("APC") for Open Access articles. Where the Submitting Author wishes to make the Work available on an Open Access basis (and intends to pay the relevant APC), the terms of reuse of such Open Access shall be governed by a Creative Commons licence – details of these licences and which Creative Commons licence will apply to this Work are set out in our licence referred to above.

Other than as permitted in any relevant BMJ Author's Self Archiving Policies, I confirm this Work has not been accepted for publication elsewhere, is not being considered for publication elsewhere and does not duplicate material already published. I confirm all authors consent to publication of this Work and authorise the granting of this licence.

For peer review only

History of heart failure and chronic kidney disease and risk of all-cause death after COVID-19 during the first three waves of the pandemic in comparison with influenza outbreaks in Sweden: a registry-based, retrospective, case-control study

Viveca Ritsinger MD PhD[1, 2], Johan Bodegard MD PhD[3], Robin Kristófi MD[4], Marcus Thuresson PhD[5], David Nathanson MD PhD[6], Thomas Nyström MD PhD[7], Jan W Eriksson MD PhD[4], Anna Norhammar MD PhD[1, 8]

1. Cardiology Unit, Department of Medicine, Solna, Karolinska Institute, Stockholm, Sweden.
2. Department of Research and Development, Region Kronoberg, Växjö, Sweden.
3. Cardiovascular, Renal and Metabolism, Medical Department, BioPharmaceuticals, AstraZeneca Nordic, Oslo, Norway.
4. Department of Medical Sciences, Clinical Diabetes and Metabolism, Uppsala University, Uppsala, Sweden.
5. Statisticon AB, Uppsala, Sweden.
6. Department of Medicine, Huddinge, Karolinska Institute, Stockholm, Sweden.
7. Department of Clinical Science and Education, Karolinska Institute, Södersjukhuset, Stockholm, Sweden.
8. Capio S:t Göran Hospital, Stockholm, Sweden.

Correspondence to:

Viveca Ritsinger

Cardiology Unit, Department of Medicine, Karolinska Institute, Stockholm, Sweden

E-mail: viveca.ritsinger@ki.se

Abstract

Objectives: To explore how cardiorenal disease (CRD; heart failure and/or chronic kidney disease) impacted mortality in men and women hospitalised for COVID-19 during the first three waves of the pandemic in Sweden in comparison to previous influenza outbreaks.

For peer review only - http://bmjopen.bmj.com/site/about/guidelines.xhtml

For peer review only

Design: A registry-based, retrospective, case-control study.

Setting: Hospital care in Sweden.

Participants: All patients in Sweden with a main hospital diagnosis of COVID-19 (January 2020-September 2021) or influenza (January 2015-December 2019) with previous CRD were identified in registries and compared with a reference group free from CRD but with COVID-19 or influenza.

Primary outcome measure: Associated risk of all-cause death during the first year was analysed using adjusted Cox proportional hazards models.

Results: In COVID-19 patients with and without prior history of CRD (n=44 866) mean age was 79.8 years (SD 11.8) and 43% were women. In influenza patients (n=8897), mean age was 80.6 years (SD 11.5) and 45% were women. COVID-19 versus influenza was associated with higher mortality risk during the first two COVID-19 waves (HR 1.53; 95% CI 1.45-1.62, $p<0.001$ and 1.52; 1.44-1.61, $p<0.001$), but not in the third wave (1.07; 0.99-1.14, $p=0.072$). CRD was an independent risk factor for all-cause death after COVID-19 in men and women (men: 1.37; 1.31-1.44, $p<0.001$; women: 1.46; 1.38-1.54, $p<0.001$). At ages <70 years, women with CRD had a similar mortality rate to men with CRD, while at ages ≥70 years, the mortality rate was higher in men.

Conclusions: Outcome after COVID-19 is worse if CRD is present. In women at ages <70 years, the presence of CRD attenuates the protective effect of female sex. COVID-19 was associated with higher mortality risk than influenza during the first two pandemic waves.

For peer review only - http://bmjopen.bmj.com/site/about/guidelines.xhtml

Strengths and limitations of this study

- This study includes all patients with cardiorenal disease hospitalised for COVID-19 during the first three pandemic waves in Sweden.
- This study compares mortality associated with COVID-19 to previous influenza outbreaks.
- Sex-based analyses by age group were performed to explore the importance of sex as a risk factor.
- This was a national registry-based study that lacked data regarding the proportion of patients vaccinated for influenza.
- The study also lacked information on laboratory analyses and variables such as body-mass index and type of heart failure.

Introduction

Infection with SARS-CoV-2 (COVID-19) globally affects people differently with some individuals more severely affected in need of hospitalisation and in more unfortunate circumstances, causing early mortality and reduced life expectancy. Starting rapidly during the first months after the initial reports from the outbreak in China [1, 2], and thereafter, there are multiple reports on COVID-19 disease course and consequences, treatment aspects, pathophysiology and characteristics of high-risk individuals. Early on it was reported that risk factors associated with hospitalisation and mortality from COVID-19 were characteristics such as advanced age, male sex, hypertension, cardiovascular disease, chronic kidney disease (CKD) and obesity. [3-7] Recently asthma and Down's syndrome were added to this list [8], while diabetes has been reported as a risk factor in some studies, while other studies found this risk to be attributed to other comorbidities. [9-12] Due to the fact that co-existence of risk

For peer review only - http://bmjopen.bmj.com/site/about/guidelines.xhtml

For peer review only

factors and multiple diseases often cluster at more advances ages, earlier reports due to low numbers and limited information, could not clarify independent associations. Some reports have been further limited to data on patients with COVID-19 admitted to intensive care, thus possibly introducing selection bias due to aspects on national and local resources and recommendations for such care, including only individuals judged to be in favour of intensive care. [7] Therefore, there is a need of more extensive and deeper analysis on contributing risk factors for severe COVID-19 outcomes in a less selected cohort to enhance present knowledge on important risk factors. Such information is of importance for improving future preventive measures. In the present study, we aimed to explore how history of heart failure and CKD (i.e., diseases that are notably interrelated and with high risk of cardiovascular and all-cause death [13]), impacts the course from COVID-19 infection and subsequent mortality during the first three waves of COVID-19 in Sweden, the only western country that did not apply a lockdown during the first pandemic year. Further, we aimed to explore this risk in comparison to previous influenza outbreaks and with a sex perspective.

For peer review only

Methods

Study design

A registry-based, retrospective, case-control study.

Data sources

Sweden has a comprehensive, nationwide public healthcare system. All citizens have a unique personal identification number (person-ID), which is mandatory for all administrative purposes (including any contact with the healthcare system and drug dispensaries), thus providing a complete medical history from a population perspective. This study included data from Statistics Sweden, National Prescribed Drug Register, the National Cause of Death

For peer review only - http://bmjopen.bmj.com/site/about/guidelines.xhtml

Register and the National Patient Register covering all hospitalisations with discharge diagnoses and all out-patient hospital visits. Individual patient-level data from the national registers were linked using the person-ID by the National Board of Health and Welfare. The linked anonymised database was managed separately by Statisticon AB, Uppsala, Sweden.

COVID-19 and influenza population

The study population includes all patients with CRD from the above specified CARE-19 cohort with a main inpatient hospital diagnosis of COVID-19 (January 1, 2020 to September 9, 2021) or influenza (January 1, 2015 - December 31, 2019). Thus, the study population includes all CRD patients hospitalised for COVID-19 in Sweden and around 2/3 of the total hospitalisations for COVID-19. Of note, the reference group is not chosen based on the COVID-19 or influenza date, but on the CRD date, as described below.

Study population

All patients from the CARE-19 cohort above 18 years of age with a main inpatient hospital diagnosis of COVID-19 (January 1, 2020 to September 9, 2021) or influenza (January 1, 2015 - December 31, 2019) were identified in the National Patient Register.

COVID-19 and influenza hospitalised patients were identified with any of the following codes as main diagnosis: B34.2, B97.2, J09.9, J10-J11, J20.8, U04.9, U07.1, U07.2 or ZV100 (ZV100 is a code used in Sweden in particularly during the initial waves of the pandemic for COVID-19 and later on more for procedures related to COVID-19). Furthermore, patients are only included once in each group: at the first COVID-19 or influenza hospitalisation. However, a prior influenza hospitalisation did not exclude the patient if later hospitalised with COVID-19.

For peer review only - http://bmjopen.bmj.com/site/about/guidelines.xhtml

CARE-19 cohort database

This database includes all patients hospitalised with heart failure and/or CKD as a main diagnosis recorded in national registries in Sweden since 1995 to September 9, 2021 that also were alive on January 1, 2010. For these patients a reference group without CRD was collected. This reference group was matched by age and sex with cases at the time point for CRD if diagnosed between the years 2010-2020, if diagnosed before 2010 then January 1, 2010 was used as time point for collecting references. Patients with heart failure were defined having at least one hospital diagnosis of heart failure: ICD-10 code I50, I11.0, I13.0, I13.2 and patients with CKD were defined as having at least one hospital diagnosis of CKD: N17-N19, I12.0-I12.9, I13.1, I13.2, N08.3, E10.2, E11.2, E12.2, E13.2, E14.2, Z49, Z99.2.

Study period

Index date was defined as the date of COVID-19 or influenza hospital admission, which is the reference date for all analyses. Baseline comorbidities were searched based on all hospital data from 1997 when ICD-10 was introduced until the day prior to index, and baseline medications were searched during one year prior to index date until the day prior to index.

Follow-up for events started at the index date (including) and ended at one year, or at date of death or end of follow-up defined as December 31, 2019 for influenza patients and September 9, 2021 for COVID-19 patients.

Statistical methods

Description of baseline characteristics was performed using mean and standard deviation for numerical variables, except for duration of first hospital stay which is described as median and interquartile range. Categorical variables are presented as number and percent. Baseline tables are stratified by type of diagnosis (COVID-19 or influenza) and CRD status (CRD/without CRD). The cumulative risk of all-cause death is presented in Kaplan-Meier

For peer review only - http://bmjopen.bmj.com/site/about/guidelines.xhtml

curves stratified by type of diagnosis (COVID-19 or influenza) and CRD status (CRD/without CRD).

The relative risk of all-cause death was analysed using a multivariable Cox regression model where the model in addition to COVID-19 vs. influenza and CRD vs. no CRD also included the following variables; age, sex, use of renin-angiotensin system inhibitors (RAAS inhibitors), coronary artery disease, stroke, peripheral artery disease (PAD), diabetes, pneumonia, chronic obstructive pulmonary disease, vitamin D-deficiency, corticosteroids, paracetamol and diagnosed obesity.

To explore the importance of risk factors relative to sex, separate analyses were in addition performed within men and women and by age group.

An exploratory analysis was performed to evaluate if the risk of all-cause death has changed during the cause of the COVID-19 pandemic period where the COVID-19 inclusion period was split into three-time intervals separated when the frequency of hospitalisations was low. Thus, the time intervals were intended to capture the first three waves of the pandemic in Sweden. The time intervals used: Wave 1: Jan 1, 2020 to August 31, 2020; Wave 2: September 1, 2020 to January 31, 2021 and Wave 3: February 1, 2021 to September 9, 2021. To limit complexity, the analysis was only performed within the CRD diagnosed patients. The cumulative incidence of all-cause death by wave are presented in Kaplan-Meier curves with Influenza as reference. In addition, as the ICD-10 code ZV100 was only used in Sweden a sensitivity analysis was performed excluding patients with a main diagnosis of ZV100.

All output from the statistical models is presented with 95 % confidence intervals and p-values. No adjustment for multiplicity has been performed.

Patient and public involvement

None.

For peer review only - http://bmjopen.bmj.com/site/about/guidelines.xhtml

Results

Characteristics of COVID-19 patients with heart failure and/or CKD

In all, 44 866 patients hospitalised with a main diagnosis of COVID-19 were identified. Of those, 23 649 had a prior history of heart failure, 11 313 had a prior history of CKD, 5767 had a prior history of the combination of heart failure and CKD and 15 671 references were free from previous CRD. Baseline characteristics for patients are presented in Table 1.

For peer review only

Table 1. Baseline characteristics in patients with COVID-19 or influenza with and without cardiorenal disease (CRD)

	COVID-19 with CRD n=29195	COVID-19 without CRD n=15671	Influenza with CRD n=5983	Influenza without CRD n=2914
Age, years, mean (SD)	79.0 (11.9)	81.2 (11.3)	79.5 (12.3)	83.1 (9.4)
Age, years, median (SD)	81 (18-107)	83 (18-106)	82 (20-106)	84 (24-107)
Females, n (%)	12474 (43)	7001 (45)	2700 (45)	1276 (44)
Year				
2020, n (%)	19729 (68)	9914 (63)	0 (0)	0 (0)
2021, n (%)	9466 (32)	5757 (37)	0 (0)	0 (0)
2015, n (%)	0 (0)	0 (0)	946 (16)	443 (15)
2016, n (%)	0 (0)	0 (0)	861 (14)	374 (13)
2017, n (%)	0 (0)	0 (0)	1154 (19)	604 (21)
2018, n (%)	0 (0)	0 (0)	1890 (32)	963 (33)
2019, n (%)	0 (0)	0 (0)	1132 (19)	530 (18)
In-hospital stay, days, median (IQR)	6 (4-11)	6 (4-10)	6 (4-10)	6 (4-10)
Heart failure, n (%)	23649 (81)	0 (0)	4971 (83)	0 (0)
Chronic kidney disease (CKD), n (%)	11313 (39)	0 (0)	2026 (34)	0 (0)
Dialysis, n (%)	1777 (6)	0 (0)	520 (9)	0 (0)
Coronary artery disease, n (%)	14314 (49)	3605 (23)	3193 (53)	786 (27)
Stroke, n (%)	8149 (28)	3655 (23)	1691 (28)	732 (25)
Atrial fibrillation, n (%)	15517 (53)	3178 (20)	3177 (53)	645 (22)
Peripheral artery disease, n (%)	3567 (12)	819 (5)	681 (11)	158 (5)
Diabetes, n (%)	10050 (34)	2974 (19)	1843 (31)	580 (20)
Chronic obstructive pulmonary disease (COPD), n (%)	6082 (21)	1421 (9)	1337 (22)	324 (11)
Any history of pneumonia (viral/bacterial), n (%)	11075 (38)	2937 (19)	2789 (47)	855 (29)
Any history of thromboembolism (DVT or PE), n (%)	3944 (14)	1407 (9)	792 (13)	270 (9)
Vitamin-D deficiency, n (%)	5780 (20)	1114 (7)	1084 (18)	156 (5)
Cancer, n (%)	10949 (38)	5780 (37)	2081 (35)	1014 (35)
Any of ACEi/ARB/MRA/ARNi/SGLT2i/β-blocker, n (%)*	26187 (90)	9677 (62)	5401 (90)	1863 (64)
RAAS inhibitor, n (%)	19285 (66)	7122 (45)	4073 (68)	1299 (45)
Statins, n (%)	15170 (52)	5566 (36)	2934 (49)	1028 (35)
Corticosteroids, n (%)	8512 (29)	2910 (19)	2096 (35)	699 (24)
Paracetamol, n (%)	17316 (59)	7325 (47)	3495 (58)	1384 (47)
Modulating antineoplastic and hormone drugs, n (%)	1698 (6)	962 (6)	299 (5)	145 (5)

* Any of Angiotensin-converting enzyme (ACE) inhibitors, Angiotensin II receptor blockers (ARBs), Mineralocorticoid receptor antagonists (MRA), Angiotensin receptor-neprilysin inhibitors (ARNi), Sodium-glucose Cotransporter-2 (SGLT2) inhibitors and Beta blockers.

Patients with COVID-19 and CRD also had other comorbidities as atrial fibrillation 53%, coronary artery disease 49%, cancer 38%, diabetes 34%, previous pneumonia 38%, stroke

For peer review only - http://bmjopen.bmj.com/site/about/guidelines.xhtml

28%, chronic obstructive pulmonary disease 21%, vitamin D deficiency 20%, history of thromboembolism 14% and peripheral artery disease 12%. Cardiovascular treatment prior to hospitalisation was commonly used with heart failure drug treatment in 90% (any of Angiotensin-converting enzyme [ACE] inhibitors, Angiotensin II receptor blockers [ARBs], Mineralocorticoid receptor antagonists [MRA], Angiotensin receptor-neprilysin inhibitors [ARNi], Sodium-glucose Cotransporter-2 [SGLT2] inhibitors and Beta blockers). Statins were used in 52% and corticosteroids in 29%.

Characteristics of COVID-19 patients without heart failure and/or CKD (reference group)

Conversely, in the reference group (COVID-19 without any history of heart failure or CKD) mean age was 81.2 years (SD 11.3) and 45% were women. Except for cancer the burden of other comorbidities was less compared to COVID-19 patients with CRD.

Characteristics of patients with influenza with heart failure and/or CKD

N=5983 patients with a history of CRD were hospitalised for influenza. Their mean age was 79.5 (SD 12.3) years and 45% were women. Apart from a previous history of pneumonia, reported in 47% in influenza patients with CRD compared to 38% in COVID-19 patients without CRD, patients hospitalised for influenza and CRD the years before the COVID-19 pandemic had a very similar comorbidity pattern as COVID-19 patients with CRD.

Outcome

During a mean follow-up period of 6.9 months (SD 4.3), 16% (n=7085) of the patients with COVID-19 died. All-cause death rate (death per 100 person years) for patients with COVID-

For peer review only - http://bmjopen.bmj.com/site/about/guidelines.xhtml

19 and CRD was 245 compared to 196 in references without CRD. When excluding patients with a COVID-19 main diagnosis of ZV100 (n=32 995) all-cause death rate (death per 100 person years) for patients with COVID-19 and CRD was even higher; 537 compared to 375 in references without CRD (mean follow-up time 6.1 [SD 4.5] months).

Corresponding mortality rate for influenza between the years 2015-2019 was lower (119 in those with CRD vs. 104 in those without).

Details on cumulative event rates for all-cause death in patients with COVID-19 or influenza stratified by previous CRD are presented in Figure 1. A higher death rate was seen for patients with CRD compared with references without with the highest rate in CRD patients with COVID-19. Except for CRD (1.41; 1.36-1.46, p<0.001), other risk factors associated with all-cause death in patients with COVID-19 were age (1.61; 1.58-1.64, p<0.001), previous pneumonia (1.19; 1.16-1.23, p<0.001) and diabetes (1.13; 1.09-1.17, p<0.001; Supplemental Table 1). Female sex was associated with a reduced risk of all-cause death (0.76; 0.73-0.78, p<0.001) while no association was seen for coronary artery disease (1.00; 0.97-1.03, p=0.881). In an adjusted Cox regression analysis in the total study cohort COVID-19 vs. influenza was associated with an increased risk of all-cause death (HR 1.54; 95% CI 1.47-1.61, p<0.001). When excluding patients with COVID-19 with a main diagnose of ZV100 corresponding HR (95% CI) for COVID-19 vs. influenza was 2.23 (2.12-2.34, p<0.001) while other risk factors were similar (Supplemental Table 2).

Figure 2 depicts cumulative incidence of all-cause death within patients with CRD by the three COVID-19 waves where a similar event rate was seen in the first two waves but with a significantly lower death rate in the third wave although still higher than that observed for influenza. Compared to influenza, COVID-19 was associated with an increased mortality risk in the first two waves (1.53; 1.45-1.62, p<0.001 and 1.52; 1.44-1.61, p<0.001) but not in the third wave (1.07; 0.99-1.14, p=0.072).

For peer review only - http://bmjopen.bmj.com/site/about/guidelines.xhtml

Sex specific analyses in patients with COVID-19 and influenza with CRD

Baseline characteristics for men and women are depicted in Supplemental Table 3 and 4. Mean age in men was 77.7 years (SD 11.7) and 80.9 years (SD 12.0) in women with even higher mean age in references without previous CRD (men 80.1 [SD 10.4] and women 82.6 years [SD 12.3]). Mean age in influenza patients with CRD was 78.1 (SD 11.9) years in men and 81.1 (SD 12.4) years in women.

Comorbidities in COVID-19 and influenza was equally distributed among men and women apart from coronary artery disease which was more common among men with COVID-19 and CRD (53% vs. 44% in women). Corresponding figures for influenza and CRD was 57% for men vs. 49% in women.

When analysing the cumulative incidence of all-cause death in men (Figure 3A) and women (Figure 3B) there was no major difference. In addition, predictors for a fatal outcome with COVID-19 (illustrated in a forest plot, Figure 4) were similar in both sexes with the strongest association for age (10-year increase, 1.66; 1.62-1.70, $p<0.001$ for men vs. 1.55; 1.51-1.60, $p<0.001$ for women), CRD (1.37; 1.31-1.44, $p<0.001$ in men vs. 1.46; 1.38-1.54, $p<0.001$ in women), PAD (1.23; 1.15-1.30, $p<0.001$ in men vs. 1.30; 1.21-1.40, $p<0.001$ in women), diabetes (1.10; 1.05-1.15, $p<0.001$ in men vs. 1.18; 1.11-1.24, $p<0.001$ in women) and previous pneumonia (1.19; 1.14-1.25, $p<0.001$ in men vs. 1.19; 1.13-1.25, $p<0.001$ in women). Previous treatment for hypertension and coronary artery disease, such as statins and RAAS inhibitors, were associated with reduced risk for all-cause death in both sexes.

Impact of age and sex

For peer review only - http://bmjopen.bmj.com/site/about/guidelines.xhtml

When analysing all-cause death rate in men and women in specified age groups (<60 years, 60-69 years, 70-79 years, ≥80 years) CRD vs. no CRD was associated with a consistent increased mortality rate after COVID-19 independent of sex and age (Figure 5). Men had higher mortality rate than women regardless of CRD, apart from at ages <70 years, where men and women with CRD had similar mortality rate. In COVID-19 references without CRD, women had a decreased mortality rate compared to men at all age groups.

For peer review only

Discussion

In this nationwide study including all patients with CRD hospitalised for COVID-19 during all three waves in Sweden there are several important findings.

First, hospitalisation for COVID-19 was associated with a 50% increased risk for mortality both in the CRD and the reference group compared to being hospitalised for influenza in the previous years illustrating that COVID-19 during the first waves was a more severe disease than influenza. The strongest predictor for mortality apart from age was cardiorenal disease (CRD) almost increasing the mortality risk by ~40% in patients with COVID-19. A history of CRD had a similar impact on patients hospitalised with influenza. We also identified peripheral artery disease, previous hospitalisation for pneumonia and diabetes as important risk factors for a fatal outcome after COVID-19.

Secondly, overall female sex compared to male was protective from a fatal outcome following hospitalisation for COVID-19. However, in the presence of CRD this sex difference was abolished with an equal mortality rate in ages below 70 years.

This indicates that COVID-19 preventive measures should be prioritised in the elderly but also regardless of sex in those with CRD. Furthermore our data strengthens that national strategies to prevent development of severe CRD could be of importance not only for

For peer review only - http://bmjopen.bmj.com/site/about/guidelines.xhtml

preventing health care burden from cardiovascular complications but also for reducing complications after severe infectious diseases as COVID-19 and influenza.

In Sweden, a high-income country with an older population and well-developed access to primary and secondary prevention, heart failure is present in 1-2% and chronic kidney disease in around 4-6%. [14, 15] In the Swedish population with age >70 years heart failure or chronic kidney disease is present in around 10-20%. [16] The CRD population (heart failure and/or CKD diagnosis prior to index) was compared to the reference group burdened with additional comorbidities as coronary artery disease present in 49% and diabetes 34% but when adjusting for other comorbidities CRD was independently associated with mortality.

Furthermore, a vast majority of the patients were treated with RAAS inhibitors and statins and thus considered to be on extensive pharmacological cardiovascular protection.

Hospitalisation for COVID-19 was associated with a significantly higher mortality risk during the first year after hospitalisation than that for hospitalisation for influenza during the previous years, especially during the first two waves where few had access to COVID-19 vaccine. This expands further knowledge from France on COVID-19 by Piroth el al. showing an increased risk of in-hospital mortality compared to that of influenza. [17] The lower mortality rate in the third wave may visualise the effect of COVID-19 vaccination in vulnerable patients and improved experiences on optimal medical strategies. Vaccination is of vast importance to reduce mortality due to influenza. Of note, influenza vaccination in the elderly in Sweden is around 50% where those with CRD and diabetes are prioritised and might have an even higher proportion of vaccination. [18]

As the ICD-10 code ZV100 (including procedures related to COVID-19) only has been used in Sweden, in particularly during the initial waves of the pandemic for COVID-19 and then more for procedures related to COVID-19, a sensitivity analysis was performed to enable

For peer review only - http://bmjopen.bmj.com/site/about/guidelines.xhtml

comparison with international studies. Although the number of patients was reduced, the results were robust with an even higher death rate in COVID-19 patients regardless of CRD and with a greater associated mortality risk compared to that of influenza.

In the present cohort, CRD was an independent predictor for mortality while in contrast coronary artery disease was not. One explanation for this might be the extensive use of cardiovascular preventive pharmacological therapy. Further, the last decades there has been a more successful prevention of incidence and complications from coronary artery disease, while prevention of incidence and complications from CRD has been less effective. [19-22] In addition, compared to an ischaemic event, CRD may have a diffuse disease pattern and therefore being clinically diagnosed later and in a more severe state. Previous treatments for hypertension and coronary artery disease, such as statins and RAAS inhibitors, were associated with a reduced risk for all-cause death in both sexes suggesting that well-controlled risk factors can prevent fatal outcome after COVID-19 infection. Accordingly, one could speculate that if CRD treatment could be improved and CRD being in a less severe state, outcome after a severe infection such as COVID-19 and also influenza would likely be less severe.

Unlike coronary artery disease, history of stroke and PAD were strong risk factors for a worse outcome probably indicating an underlying general severe atherosclerotic burden and frailty. Few studies have analysed the outcome of COVID-19 in patients with heart failure and/or CKD. In accordance with our results, Bergman et al [8] also found that in Sweden high age, diabetes, cardiovascular disease (however not analysing heart failure specifically), hypertension, renal failure and previous pneumonia were associated with all-cause death in hospitalised and non-hospitalised COVID-19 cases although with a shorter median follow-up time than ours (four months).

For peer review only - http://bmjopen.bmj.com/site/about/guidelines.xhtml

Interestingly, we found that history of hospitalisation for pneumonia was common among COVID-19 patients, in particular, those with CRD (present in 39%), which was higher than previously reported by Bergman et al [8] (15%). Also, in the influenza population with CRD previous pneumonia was common (47%). After adjusting for age and other risk factors this was also one of the most important predictors for a fatal outcome. This does not seem to be explained by presence of chronic obstructive pulmonary disease (COPD) which was less common (present in around one fifth) and also a less important risk factor. Rather this seems to indicate a patient who is predisposed to a more severe disease development after a pulmonary infection. Our data are in line with a recent report from Ballin et al who analysed 30-day mortality in long-term care facility residents in Sweden and also identified previous pneumonia as an important risk factor for mortality in those diagnosed with COVID-19. [23]

Another Swedish study has reported on the selected group admitted to ICU and found diabetes, hypertension and obesity as independently associated risk factors for severe COVID-19, when having access to hospital recorded variables in the Swedish Intensive Care Registry (SIR). [7] In the present study, obesity diagnosis (reported in the National Patient Register) was not a predictor for mortality which may reflect "*the obesity paradox"* where overweight in heart failure and in chronic kidney disease may indicate a less catabolic patient. [24] However, this should be interpreted with caution as we have no information about body-mass index (BMI) and the reporting of obesity in the National Patient Register is of uncertain representativeness.

In CRD patients hospitalised for COVID-19, almost half of the patients were women. Although significantly older, female sex was protective in survival analysis. However, in the presence of CRD this sex difference was abolished. At ages below 70 years, men and women had similar mortality rates but at ages above 70 men had a higher mortality rate than women. This pattern was not seen in the reference group where women had a lower mortality rate than

For peer review only - http://bmjopen.bmj.com/site/about/guidelines.xhtml

men at all ages. Thus, in the presence of CRD the protective effect of female sex was not present. Indeed, within women hospitalised for COVID-19 a history of CRD was identified as being the strongest risk factor for a fatal outcome. Presence of coronary artery disease, although less common in women, and previous hospitalisation for pneumonia were other risk factors for a fatal outcome in women with COVID-19.

The major strength of this study is the use of national registries enabling inclusion of all CRD patients hospitalised for COVID-19 during all three waves in Sweden. Furthermore, we had the ability to compare COVID-19 with characteristics and outcome from previous influenza hospitalisations showing a higher mortality rate from covid-19 when vaccination coverage still was low. Another strength is the comparison with a reference group with similar age and sex structure apart from CRD. A limitation is the inclusion criteria was restricted to hospitalised COVID-19 patients where patients that died with COVID-19 outside hospital care, as in elderly home care, have been missed. This is also true for the influenza cohort. Further, we have no individual data on influenza vaccination or proportion of patients vaccinated for influenza. Another limitation is the lack of information on laboratory analysis or variables as BMI as these registries do not contain such information. For example, obesity diagnoses are captured only from the National Patient Register and therefore associations between BMI and mortality could not be analysed in this study. However, in real-life situations risk factor from medical history is often the only risk evaluation when prioritising vaccination (as in this report) and seldom based on HbA1c or creatinine levels.

Conclusion

We demonstrate that outcome in patients with COVID-19 and influenza is worse if prior CRD is present independently of age and sex. COVID-19 was associated with higher mortality risk

For peer review only - http://bmjopen.bmj.com/site/about/guidelines.xhtml

than influenza during the first two waves. Furthermore, the presence of CRD abolished the protective effect of female sex in women at ages below 70 years. This study highlights the importance of preventing heart failure and chronic kidney disease in the general population to reduce mortality from severe infections in the future.

Contributors

The study design was developed by J.B. and M.T. M.T. performed the statistical analyses and managed the database. V.R. and A.N. finalised the manuscript after adjustments by J.B., M.T., J.W.E., R.K., T.N. and D.N. All authors made a substantial contribution to this article and was involved in the interpretation of the results.

Competing interests

The authors declared the following potential conflicts of interest with respect to the research, authorship and publication of this article: V.R. has received honoraria on expert group participation from Astra Zeneca, Novo Nordisk and Boehringer Ingelheim. A.N. has received honoraria from Astra Zeneca, Merck Sharp & Dohme, Eli Lilly and Company, Novo Nordisk and Boehringer Ingelheim on expert group participation. T.N. has received unrestricted grants from AstraZeneca and NovoNordisk and has served on national advisory boards of Abbot, Amgen, Novo Nordisk, Sanofi-Aventis, Eli Lilly, MSD and Boehringer Ingelheim. J.W.E. has received research support or honoraria from AstraZeneca, NovoNordisk, Bayer, Ilya Pharma, Merck-Sharp & Dohme, Boehringer-Ingelheim. R.K. and D.N. reports no conflicts of interest. J.B. holds a full-time position at AstraZeneca as an epidemiologist. M.T. holds a full-time position by an independent statistical consultant company, Statisticon AB, Uppsala, Sweden, of which AstraZeneca Nordic is a client.

For peer review only - http://bmjopen.bmj.com/site/about/guidelines.xhtml

For peer review only

Funding

The study was fully sponsored by AstraZeneca. V.R. has received funding from the Department of Research and Development Region Kronoberg and the Family Kamprad Foundation. A.N. has received funding from the Family Kamprad Foundation and from Capio S:t Görans hospital.

Acknowledgements

The authors are grateful to Susanna Jerström and Helena Goike at AstraZeneca for logistic support and valuable comments on the manuscript. Urban Olsson, Statisticon AB, is acknowledged for database management.

Ethics approval

The study was approved by the Stockholm Regional Ethics Committee (reference numbers 2020-05714 and 2013/2206-31). Ethical permission approved a reference group to a collected population with cardiorenal disease (CRD; heart failure and/or chronic kidney disease [CARE-19]) but not a reference group to COVID-19 or influenza patients.

Data availability statement

No additional data available.

For peer review only - http://bmjopen.bmj.com/site/about/guidelines.xhtml

References

1. Phelan AL, Katz R, Gostin LO. The Novel Coronavirus Originating in Wuhan, China: Challenges for Global Health Governance. *JAMA* 2020;323(8):709-10.

2. Guan WJ, Ni ZY, Hu Y, Liang WH, Ou CQ, He JX, Liu L, Shan H, Lei CL, Hui DSC, Du B, Li LJ, Zeng G, Yuen KY, Chen RC, Tang CL, Wang T, Chen PY, Xiang J, Li SY, Wang JL, Liang ZJ, Peng YX, Wei L, Liu Y, Hu YH, Peng P, Wang JM, Liu JY, Chen Z, Li G, Zheng ZJ, Qiu SQ, Luo J, Ye CJ, Zhu SY, Zhong NS; China Medical Treatment Expert Group for Covid-19. Clinical Characteristics of Coronavirus Disease 2019 in China. *N Engl J Med* 2020;382(18):1708-20.

3. Grasselli G, Greco M, Zanella A, Albano G, Antonelli M, Bellani G, Bonanomi E, Cabrini L, Carlesso E, Castelli G, Cattaneo S, Cereda D, Colombo S, Coluccello A, Crescini G, Forastieri Molinari A, Foti G, Fumagalli R, Iotti GA, Langer T, Latronico N, Lorini FL, Mojoli F, Natalini G, Pessina CM, Ranieri VM, Rech R, Scudeller L, Rosano A, Storti E, Thompson BT, Tirani M, Villani PG, Pesenti A, Cecconi M; COVID-19 Lombardy ICU Network. Risk Factors Associated With Mortality Among Patients With COVID-19 in Intensive Care Units in Lombardy, Italy. *JAMA Intern Med* 2020;180(10):1345-55.

4. Evans D, Inglesby P, Cockburn J, McDonald HI, MacKenna B, Tomlinson L, Douglas IJ, Rentsch CT, Mathur R, Wong AYS, Grieve R, Harrison D, Forbes H, Schultze A, Croker R, Parry J, Hester F, Harper S, Perera R, Evans SJW, Smeeth L, Goldacre B. Factors associated with COVID-19-related death using OpenSAFELY. *Nature* 2020;584(7821):430-6.

5. Reilev M, Kristensen KB, Pottegård A, Lund LC, Hallas J, Ernst MT, Christiansen CF, Sørensen HT, Johansen NB, Brun NC, Voldstedlund M, Støvring H, Thomsen MK, Christensen S, Gubbels S, Krause TG, Mølbak K, Thomsen RW. Characteristics and

For peer review only

For peer review only - http://bmjopen.bmj.com/site/about/guidelines.xhtml

predictors of hospitalization and death in the first 11 122 cases with a positive RT-PCR test for SARS-CoV-2 in Denmark: a nationwide cohort. *Int J Epidemiol* 2020;49(5):1468-81.

6. Cummings MJ, Baldwin MR, Abrams D, Jacobson SD, Meyer BJ, Balough EM, Aaron JG, Claassen J, Rabbani LE, Hastie J, Hochman BR, Salazar-Schicchi J, Yip NH, Brodie D, O'Donnell MR. Epidemiology, clinical course, and outcomes of critically ill adults with COVID-19 in New York City: a prospective cohort study. *Lancet* 2020;395(10239):1763-70.

7. Svensson P, Hofmann R, Häbel H, Jernberg T, Nordberg P. Association between cardiometabolic disease and severe COVID-19: a nationwide case-control study of patients requiring invasive mechanical ventilation. *BMJ Open* 2021;11(2):e044486.

8. Bergman J, Ballin M, Nordström A, Nordström P. Risk factors for COVID-19 diagnosis, hospitalization, and subsequent all-cause mortality in Sweden: a nationwide study. *Eur J Epidemiol* 2021;36(3):287-98.

9. Al-Salameh A, Lanoix JP, Bennis Y, Andrejak C, Brochot E, Deschasse G, Dupont H, Goeb V, Jaureguy M, Lion S, Maizel J, Moyet J, Vaysse B, Desailloud R, Ganry O, Schmit JL, Lalau JD. Characteristics and outcomes of COVID-19 in hospitalized patients with and without diabetes. *Diabetes Metab Res Rev* 2021;37(3):e3388.

10. Rawshani A, Kjölhede EA, Rawshani A, Sattar N, Eeg-Olofsson K, Adiels M, Ludvigsson J, Lindh M, Gisslén M, Hagberg E, Lappas G, Eliasson B, Rosengren A. Severe COVID-19 in people with type 1 and type 2 diabetes in Sweden: A nationwide retrospective cohort study. *Lancet Reg Health Eur* 2021;4:100105.

11. Barron E, Bakhai C, Kar P, Weaver A, Bradley D, Ismail H, Knighton P, Holman N, Khunti K, Sattar N, Wareham NJ, Young B, Valabhji J. Associations of type 1 and type 2 diabetes with COVID-19-related mortality in England: a whole-population study. *Lancet Diabetes Endocrinol* 2020;8(10):813-22.

For peer review only

For peer review only - http://bmjopen.bmj.com/site/about/guidelines.xhtml

12. Holman N, Knighton P, Kar P, O'Keefe J, Curley M, Weaver A, Barron E, Bakhai C, Khunti K, Wareham NJ, Sattar N, Young B, Valabhji J. Risk factors for COVID-19-related mortality in people with type 1 and type 2 diabetes in England: a population-based cohort study. *Lancet Diabetes Endocrinol* 2020;8(10):823-33.

13. Rangaswami J, Bhalla V, Blair JEA, Chang TI, Costa S, Lentine KL, Lerma EV, Mezue K, Molitch M, Mullens W, Ronco C, Tang WHW, McCullough PA; American Heart Association Council on the Kidney in Cardiovascular Disease and Council on Clinical Cardiology. Cardiorenal Syndrome: Classification, Pathophysiology, Diagnosis, and Treatment Strategies: A Scientific Statement From the American Heart Association. *Circulation* 2019;139(16):e840-e878.

14. Seferović PM, Vardas P, Jankowska EA, Maggioni AP, Timmis A, Milinković I, Polovina M, Gale CP, Lund LH, Lopatin Y, Lainscak M, Savarese G, Huculeci R, Kazakiewicz D, Coats AJS; National Heart Failure Societies of the ESC member countries (see Appendix). The Heart Failure Association Atlas: Heart Failure Epidemiology and Management Statistics 2019. *Eur J Heart Fail* 2021;23(6):906-14.

15. Zarrinkoub R, Wettermark B, Wändell P, Mejhert M, Szulkin R, Ljunggren G, Kahan T. The epidemiology of heart failure, based on data for 2.1 million inhabitants in Sweden. *Eur J Heart Fail* 2013;15(9):995-1002.

16. Gasparini A, Evans M, Coresh J, Grams ME, Norin O, Qureshi AR, Runesson B, Barany P, Ärnlöv J, Jernberg T, Wettermark B, Elinder CG, Carrero JJ. Prevalence and recognition of chronic kidney disease in Stockholm healthcare. *Nephrol Dial Transplant* 2016;31(12):2086-94.

17. Piroth L, Cottenet J, Mariet AS, Bonniaud P, Blot M, Tubert-Bitter P, Quantin C. Comparison of the characteristics, morbidity, and mortality of COVID-19 and seasonal

For peer review only

influenza: a nationwide, population-based retrospective cohort study. *Lancet Respir Med* 2021;9(3):251-9.

18. The Public Health Agency of Sweden. Available from: https://www.folkhalsomyndigheten.se/folkhalsorapportering-statistik/statistikdatabaser-och-visualisering/vaccinationsstatistik/statistik-for-influensavaccinationer/. Date of access 10 November, 2021.

19. Yang D, Dzayee DA, Beiki O, de Faire U, Alfredsson L, Moradi T. Incidence and case fatality after day 28 of first time myocardial infarction in Sweden 1987-2008. *Eur J Prev Cardiol* 2012;19(6):1304-15.

20. Szummer K, Wallentin L, Lindhagen L, Alfredsson J, Erlinge D, Held C, James S, Kellerth T, Lindahl B, Ravn-Fischer A, Rydberg E, Yndigegn T, Jernberg T. Improved outcomes in patients with ST-elevation myocardial infarction during the last 20 years are related to implementation of evidence-based treatments: experiences from the SWEDEHEART registry 1995-2014. *Eur Heart J* 2017;38(41):3056-65.

21. Szummer K, Wallentin L, Lindhagen L, Alfredsson J, Erlinge D, Held C, James S, Kellerth T, Lindahl B, Ravn-Fischer A, Rydberg E, Yndigegn T, Jernberg T. Relations between implementation of new treatments and improved outcomes in patients with non-ST-elevation myocardial infarction during the last 20 years: experiences from SWEDEHEART registry 1995 to 2014. *Eur Heart J* 2018;39(42):3766-76.

22. House AA, Wanner C, Sarnak MJ, Piña IL, McIntyre CW, Komenda P, Kasiske BL, Deswal A, deFilippi CR, Cleland JGF, Anker SD, Herzog CA, Cheung M, Wheeler DC, Winkelmayer WC, McCullough PA; Conference Participants. Heart failure in chronic kidney disease: conclusions from a Kidney Disease: Improving Global Outcomes (KDIGO) Controversies Conference. *Kidney Int* 2019;95(6):1304-17.

For peer review only

For peer review only - http://bmjopen.bmj.com/site/about/guidelines.xhtml

23. Ballin M, Bergman J, Kivipelto M, Nordström A, Nordström P. Excess Mortality After COVID-19 in Swedish Long-Term Care Facilities. *J Am Med Dir Assoc* 2021;22(8):1574-80.

24. Ritsinger V, Brismar K, Malmberg K, Mellbin L, Näsman P, Rydén L, Söderberg S, Tenerz Å, Norhammar A. Elevated levels of adipokines predict outcome after acute myocardial infarction - a long-term follow up of the GAMI cohort. *Diab Vasc Dis Res* 2017;14(2):77-87.

For peer review only

For peer review only - http://bmjopen.bmj.com/site/about/guidelines.xhtml

Figure legends

Figure 1. Cumulative incidence of all-cause death in COVID-19 patients between the years 2020-2021 with cardiorenal disease (CRD; blue) and without CRD (dotted blue) and in influenza patients between the years 2015-2019 with CRD (green) and without CRD (dotted green)

Figure 2. All-cause death in patients with cardiorenal disease and COVID-19 or influenza by the three COVID-19 waves in Sweden

Figure 3. Cumulative incidence of all-cause death in men (A) and women (B) with COVID-19 between the years 2020 and 2021 with cardiorenal disease (CRD; blue) and without CRD (dotted blue) and in men (A) and women (B) with influenza between the years 2015 and 2019 with CRD (green) and without CRD (dotted green)

Figure 4. Adjusted associated HR (95% CI) for all-cause death in men and women and mortality associated with COVID-19 vs influenza

Figure 5. All-cause death rate by sex and age groups stratified by cardiorenal disease (CRD) in patients with COVID-19

For peer review only

For peer review only - http://bmjopen.bmj.com/site/about/guidelines.xhtml

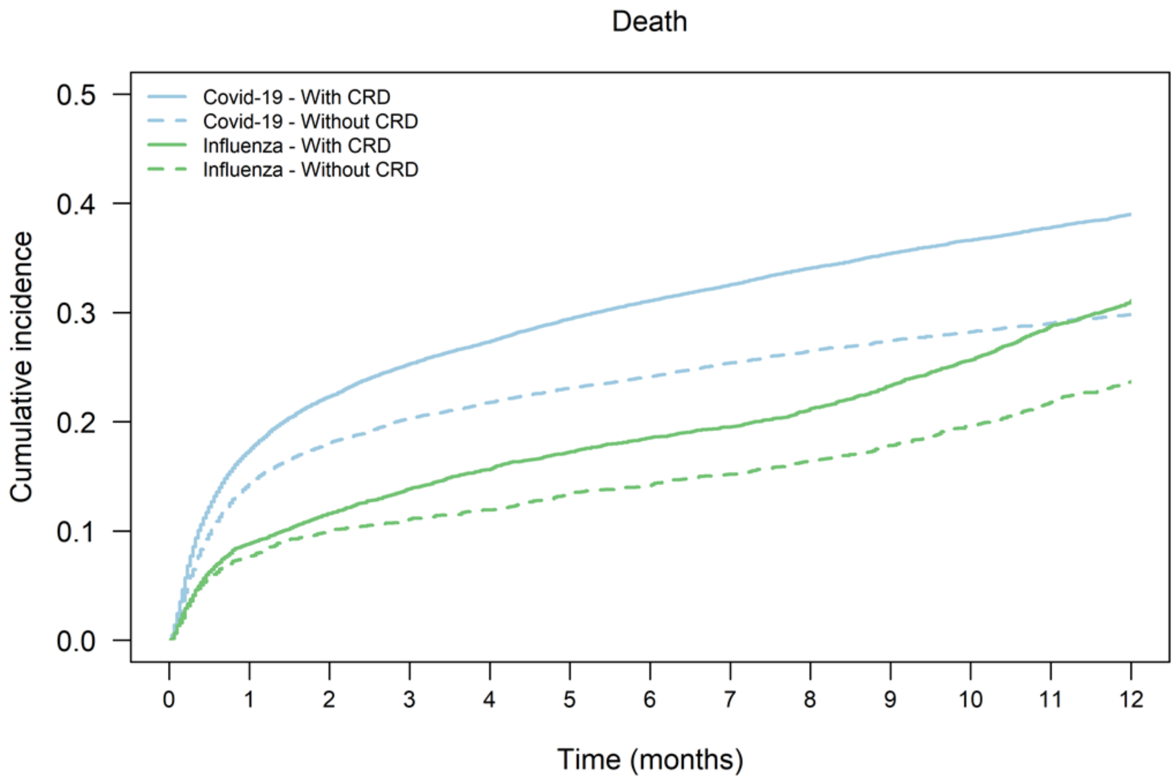
Death
Covid-19 - With CRD
Covid-19 - Without CRD
Influenza - With CRD
Influenza - Without CRD
Cumulative incidence
0.0
0.1
0.2
0.3
0.4
0.5
0 1 2 3 4 5 6 7 8 9 10 11 12
Time (months)

review only

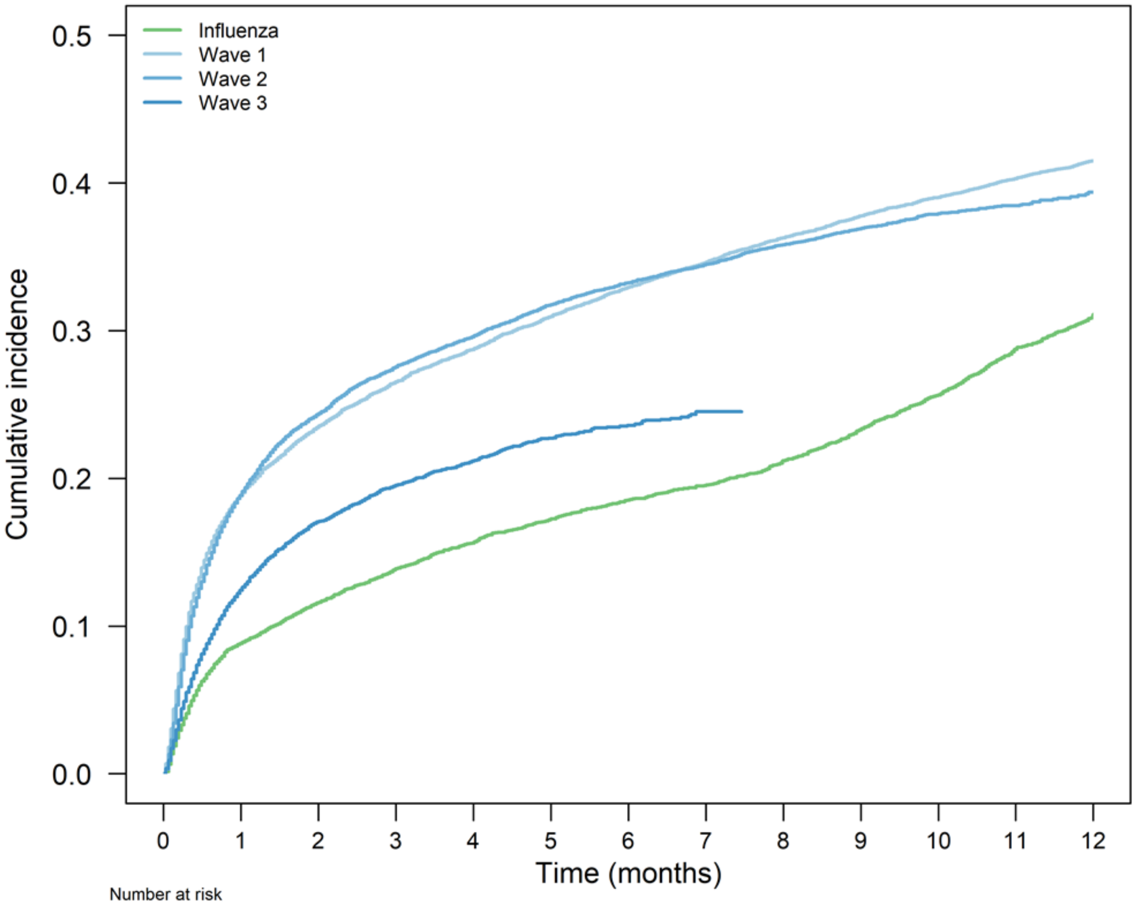

Number at risk

	0	2	4	6	8	10	12
Influenza	5983	5243	5003	4829	4655	4175	3529
Wave 1	10918	8360	7783	7324	6957	6657	6386
Wave 2	11422	8646	8045	7626	6546	2596	439
Wave 3	6855	5583	3990	1704			

A.

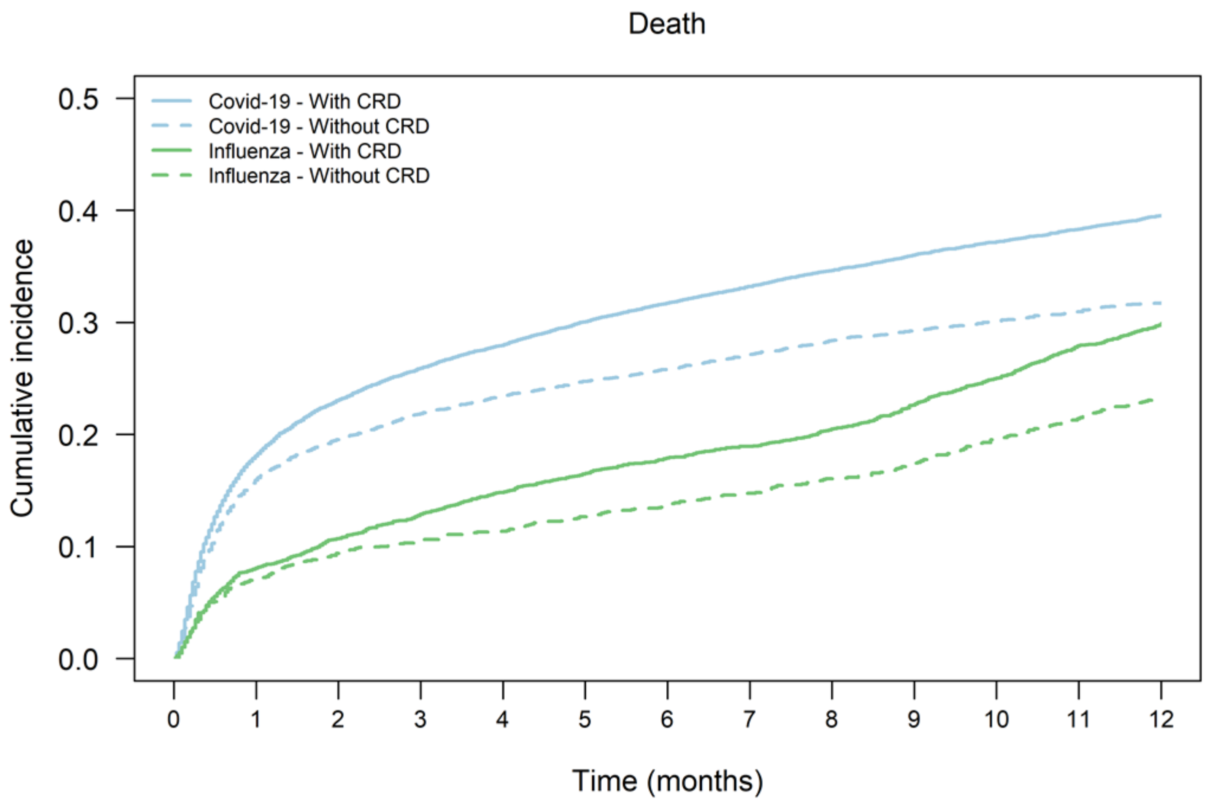
Death
Covid-19 - With CRD
Covid-19 - Without CRD
Influenza - With CRD
Influenza - Without CRD
Cumulative incidence
0.0
0.1
0.2
0.3
0.4
0.5
0
1
2
3
4
5
6
7
8
9
10
11
12
Time (months)

B.

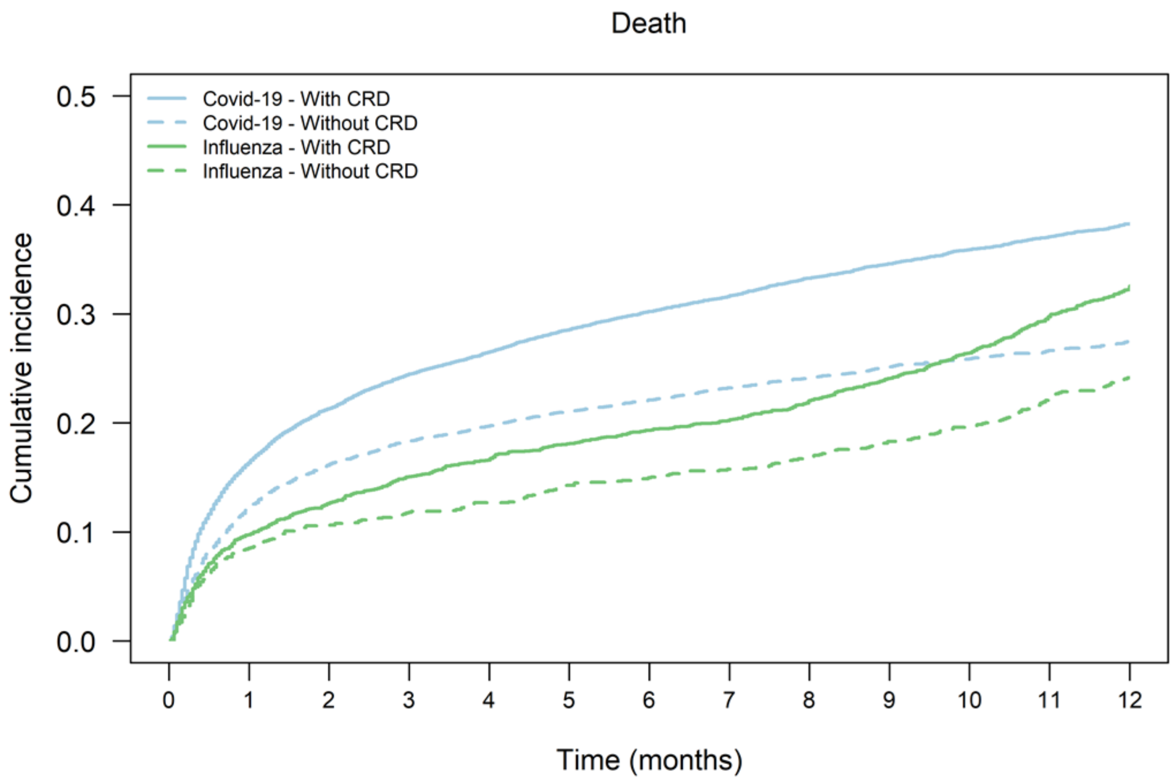
Death
Covid-19 - With CRD
Covid-19 - Without CRD
Influenza - With CRD
Influenza - Without CRD
Cumulative incidence
0.0
0.1
0.2
0.3
0.4
0.5
0
1
2
3
4
5
6
7
8
9
10
11
12
Time (months)

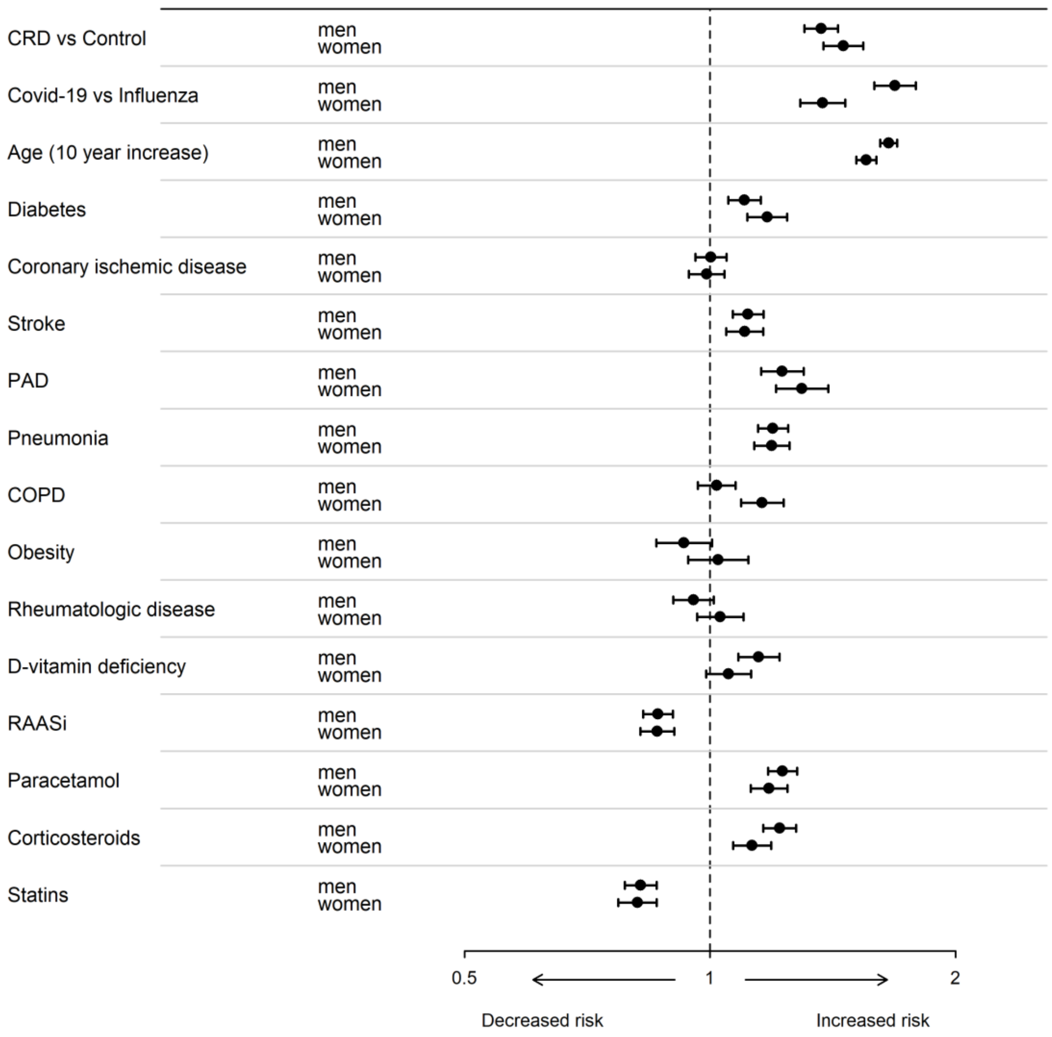
CRD vs Control
Covid-19 vs Influenza
Age (10 year increase)
Diabetes
Coronary ischemic disease
Stroke
PAD
Pneumonia
COPD
Obesity
Rheumatologic disease
D-vitamin deficiency
RAASi
Paracetamol
Corticosteroids
Statins
men
women
0.5
1
2
Decreased risk
Increased risk

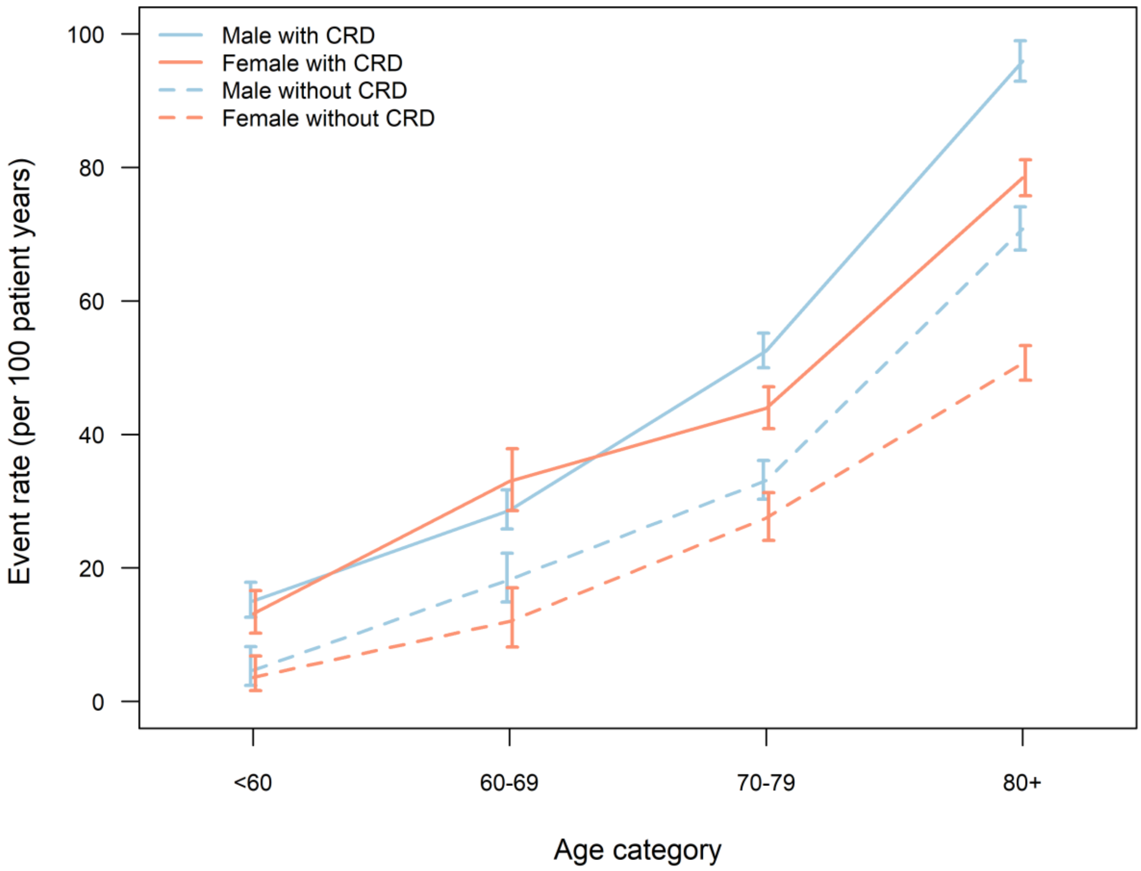

For peer review only

Supplemental material

Table 1. Adjusted HR and 95% CI for all-cause death in COVID-19 patients.

	Hazard ratio	95% CI	P-value
Cardiorenal disease (CRD) vs. no CRD	1.41	(1.36-1.46)	<0.001
Age per 10 years increase	1.61	(1.58-1.64)	<0.001
Female sex	0.76	(0.73-0.78)	<0.001
Diabetes	1.13	(1.09-1.17)	<0.001
Coronary artery disease	1.00	(0.97-1.03)	0.881
Stroke	1.11	(1.07-1.15)	<0.001
Peripheral artery disease (PAD)	1.25	(1.19-1.31)	<0.001
Pneumonia	1.19	(1.16-1.23)	<0.001
Chronic obstructive pulmonary disease (COPD)	1.08	(1.04-1.13)	<0.001
Obesity	0.97	(0.92-1.03)	0.335
Rheumatologic disease	0.99	(0.95-1.03)	0.570
D-vitamin deficiency	1.10	(1.06-1.15)	<0.001
RAAS inhibitors	0.86	(0.83-0.89)	<0.001
Paracetamol	1.21	(1.17-1.25)	<0.001
Corticosteroids	1.18	(1.14-1.22)	<0.001
Statins	0.82	(0.79-0.85)	<0.001

For peer review only

For peer review only - http://bmjopen.bmj.com/site/about/guidelines.xhtml

Table 2. Adjusted HR and 95% CI for all-cause death in COVID-19 excluding patients with ZV100 as a main diagnosis.

	Hazard ratio	95% CI	P-value
Cardiorenal disease (CRD) vs. no CRD	1.43	(1.35-1.51)	<0.001
Age per 10 years increase	1.70	(1.65-1.75)	<0.001
Female sex	0.77	(0.73-0.81)	<0.001
Diabetes	1.17	(1.11-1.23)	<0.001
Coronary artery disease	1.03	(0.98-1.08)	0.315
Stroke	1.17	(1.11-1.23)	<0.001
Peripheral artery disease (PAD)	1.35	(1.25-1.45)	<0.001
Pneumonia	1.19	(1.13-1.25)	<0.001
Chronic obstructive pulmonary disease (COPD)	1.07	(1.01-1.14)	0.033
Obesity	0.98	(0.89-1.07)	0.601
Rheumatologic disease	1.06	(0.99-1.13)	0.071
D-vitamin deficiency	1.09	(1.02-1.16)	0.008
RAAS inhibitors	0.88	(0.84-0.93)	<0.001
Paracetamol	1.23	(1.17-1.30)	<0.001
Corticosteroids	1.13	(1.07-1.19)	<0.001
Statins	0.82	(0.78-0.87)	<0.001

For peer review only - http://bmjopen.bmj.com/site/about/guidelines.xhtml

Table 3. Baseline characteristics in men with COVID-19 or influenza with and without cardiorenal disease (CRD).

	Covid-19 with CRD n=16721	Covid-19 without CRD n=8670	Influenza with CRD n=3283	Influenza without CRD n=1638
Age, years, mean (SD)	77.7 (11.7)	80.1 (10.4)	78.1 (11.9)	82.1 (9.1)
Age, years, median (min-max)	79 (18-103)	82 (20-103)	80 (20-106)	83 (24-104)
Females, n (%)	0 (0)	0 (0)	0 (0)	0 (0)
Year				
2020, n (%)	11329 (68)	5477 (63)	0 (0)	0 (0)
2021, n (%)	5392 (32)	3193 (37)	0 (0)	0 (0)
2015, n (%)	0 (0)	0 (0)	515 (16)	246 (15)
2016, n (%)	0 (0)	0 (0)	459 (14)	227 (14)
2017, n (%)	0 (0)	0 (0)	624 (19)	328 (20)
2018, n (%)	0 (0)	0 (0)	1051 (32)	531 (32)
2019, n (%)	0 (0)	0 (0)	634 (19)	306 (19)
In-hospital stay, days, median (IQR)	6 (4-11)	6 (4-10)	6 (4-10)	5 (3-9)
Heart failure, n (%)	13275 (79)	0 (0)	2648 (81)	0 (0)
Chronic kidney disease (CKD), n (%)	7025 (42)	0 (0)	1260 (38)	0 (0)
Dialysis, n (%)	1174 (7)	0 (0)	350 (11)	0 (0)
Coronary artery disease, n (%)	8810 (53)	2270 (26)	1882 (57)	485 (30)
Stroke, n (%)	4747 (28)	2141 (25)	967 (29)	441 (27)
Atrial fibrillation, n (%)	9102 (54)	1904 (22)	1759 (54)	379 (23)
Peripheral artery disease (PAD), n (%)	2127 (13)	488 (6)	422 (13)	86 (5)
Diabetes, n (%)	6158 (37)	1863 (21)	1104 (34)	372 (23)
Chronic obstructive pulmonary disease (COPD), n (%)	3243 (19)	772 (9)	688 (21)	161 (10)
Any history of pneumonia (viral/bacterial), n (%)	6276 (38)	1681 (19)	1519 (46)	475 (29)
Any history of thromboembolism (DVT or PE), n (%)	2120 (13)	741 (9)	399 (12)	139 (8)
Vitamin-D deficiency, n (%)	3081 (18)	450 (5)	610 (19)	70 (4)
Cancer, n (%)	6372 (38)	3300 (38)	1175 (36)	581 (35)
Any of ACEi/ARB/MRA/ARNi/SGLT2i/β-blocker, n (%)*	15056 (90)	5322 (61)	2974 (91)	1023 (62)
RAAS inhibitor, n (%)	11293 (68)	4027 (46)	2307 (70)	729 (45)
Statins, n (%)	9733 (58)	3521 (41)	1875 (57)	655 (40)
Corticosteroids, n (%)	4585 (27)	1492 (17)	1081 (33)	339 (21)
Paracetamol, n (%)	8850 (53)	3497 (40)	1667 (51)	656 (40)
Modulating antineoplastic and hormone drugs, n (%)	1182 (7)	706 (8)	210 (6)	110 (7)

* Any of Angiotensin-converting enzyme (ACE) inhibitors, Angiotensin II receptor blockers (ARBs), Mineralocorticoid receptor antagonists (MRA), Angiotensin receptor-neprilysin inhibitors (ARNi), Sodium-glucose Cotransporter-2 (SGLT2) inhibitors and Beta blockers.

For peer review only - http://bmjopen.bmj.com/site/about/guidelines.xhtml

Table 4. Baseline characteristics in women with COVID-19 or influenza with and without cardiorenal disease (CRD).

	Covid-19 with CRD n=12474	Covid-19 without CRD n=7001	Influenza with CRD n=2700	Influenza without CRD n=1276
Age, years, mean (SD)	80.9 (12.0)	82.6 (12.3)	81.1 (12.4)	84.3 (9.6)
Age, years, median (min-max)	83 (18-107)	85 (18-106)	84 (20-102)	86 (32-107)
Females, n (%)	12474 (100)	7001 (100)	2700 (100)	1276 (100)
Year				
2020, n (%)	8400 (67)	4437 (63)	0 (0)	0 (0)
2021, n (%)	4074 (33)	2564 (37)	0 (0)	0 (0)
2015, n (%)	0 (0)	0 (0)	431 (16)	197 (15)
2016, n (%)	0 (0)	0 (0)	402 (15)	147 (12)
2017, n (%)	0 (0)	0 (0)	530 (20)	276 (22)
2018, n (%)	0 (0)	0 (0)	839 (31)	432 (34)
2019, n (%)	0 (0)	0 (0)	498 (18)	224 (18)
In-hospital stay, days, median (IQR)	7 (4-11)	6 (4-10)	7 (4-11)	6 (4-11)
Heart failure, n (%)	10374 (83)	0 (0)	2323 (86)	0 (0)
Chronic kidney disease (CKD), n (%)	4288 (34)	0 (0)	766 (28)	0 (0)
Dialysis, n (%)	603 (5)	0 (0)	170 (6)	0 (0)
Coronary artery disease, n (%)	5504 (44)	1335 (19)	1311 (49)	301 (24)
Stroke, n (%)	3402 (27)	1514 (22)	724 (27)	291 (23)
Atrial fibrillation, n (%)	6415 (51)	1274 (18)	1418 (53)	266 (21)
Peripheral artery disease (PAD), n (%)	1440 (12)	331 (5)	259 (10)	72 (6)
Diabetes, n (%)	3892 (31)	1111 (16)	739 (27)	208 (16)
Chronic obstructive pulmonary disease (COPD), n (%)	2839 (23)	649 (9)	649 (24)	163 (13)
Any history of pneumonia (viral/bacterial), n (%)	4799 (38)	1256 (18)	1270 (47)	380 (30)
Any history of thromboembolism (DVT or PE), n (%)	1824 (15)	666 (10)	393 (15)	131 (10)
Vitamin-D deficiency, n (%)	2699 (22)	664 (9)	474 (18)	86 (7)
Cancer, n (%)	4577 (37)	2480 (35)	906 (34)	433 (34)
Any of ACEi/ARB/MRA/ARNi/SGLT2i/β-blocker, n (%)*	11131 (89)	4355 (62)	2427 (90)	840 (66)
RAAS inhibitor, n (%)	7992 (64)	3095 (44)	1766 (65)	570 (45)
Statins, n (%)	5437 (44)	2045 (29)	1059 (39)	373 (29)
Corticosteroids, n (%)	3927 (31)	1418 (20)	1015 (38)	360 (28)
Paracetamol, n (%)	8466 (68)	3828 (55)	1828 (68)	728 (57)
Modulating antineoplastic and hormone drugs, n (%)	516 (4)	256 (4)	89 (3)	35 (3)

* Any of Angiotensin-converting enzyme (ACE) inhibitors, Angiotensin II receptor blockers (ARBs), Mineralocorticoid receptor antagonists (MRA), Angiotensin receptor-neprilysin inhibitors (ARNi), Sodium-glucose Cotransporter-2 (SGLT2) inhibitors and Beta blockers.

For peer review only - http://bmjopen.bmj.com/site/about/guidelines.xhtml

STROBE Statement—checklist of items that should be included in reports of observational studies

	Item No	Recommendation	Page No
**Title and abstract**	1	(*a*) Indicate the study's design with a commonly used term in the title or the abstract	1
		(*b*) Provide in the abstract an informative and balanced summary of what was done and what was found	1
**Introduction**			
Background/rationale	2	Explain the scientific background and rationale for the investigation being reported	1
Objectives	3	State specific objectives, including any prespecified hypotheses	4-5
**Methods**			
Study design	4	Present key elements of study design early in the paper	5-6
Setting	5	Describe the setting, locations, and relevant dates, including periods of recruitment, exposure, follow-up, and data collection	5-7
Participants	6	(*a*) *Cohort study*—Give the eligibility criteria, and the sources and methods of selection of participants. Describe methods of follow-up<br>*Case-control study*—Give the eligibility criteria, and the sources and methods of case ascertainment and control selection. Give the rationale for the choice of cases and controls<br>*Cross-sectional study*—Give the eligibility criteria, and the sources and methods of selection of participants	5-7
		(*b*) *Cohort study*—For matched studies, give matching criteria and number of exposed and unexposed<br>*Case-control study*—For matched studies, give matching criteria and the number of controls per case	5-7
Variables	7	Clearly define all outcomes, exposures, predictors, potential confounders, and effect modifiers. Give diagnostic criteria, if applicable	5-7
Data sources/ measurement	8*	For each variable of interest, give sources of data and details of methods of assessment (measurement). Describe comparability of assessment methods if there is more than one group	*5-7*
Bias	9	Describe any efforts to address potential sources of bias	16
Study size	10	Explain how the study size was arrived at	5-6
Quantitative variables	11	Explain how quantitative variables were handled in the analyses. If applicable, describe which groupings were chosen and why	7-8
Statistical methods	12	(*a*) Describe all statistical methods, including those used to control for confounding	7-8
		(*b*) Describe any methods used to examine subgroups and interactions	7-8
		(*c*) Explain how missing data were addressed	7-8
		(*d*) *Cohort study*—If applicable, explain how loss to follow-up was addressed<br>*Case-control study*—If applicable, explain how matching of cases and controls was addressed<br>*Cross-sectional study*—If applicable, describe analytical methods taking account of sampling strategy	7-8
		(*e*) Describe any sensitivity analyses	7-8

Continued on next page

For peer review only - http://bmjopen.bmj.com/site/about/guidelines.xhtml

**Results**			
Participants	13*	(a) Report numbers of individuals at each stage of study—eg numbers potentially eligible, examined for eligibility, confirmed eligible, included in the study, completing follow-up, and analysed	5-7
		(b) Give reasons for non-participation at each stage	5-7
		(c) Consider use of a flow diagram	
Descriptive data	14*	(a) Give characteristics of study participants (eg demographic, clinical, social) and information on exposures and potential confounders	8-9
		(b) Indicate number of participants with missing data for each variable of interest	8-9
		(c) *Cohort study*—Summarise follow-up time (eg, average and total amount)	8-9
Outcome data	15*	*Cohort study*—Report numbers of outcome events or summary measures over time	
		Case-control study—Report numbers in each exposure category, or summary measures of exposure	*8-9*
		Cross-sectional study—Report numbers of outcome events or summary measures	
Main results	16	(*a*) Give unadjusted estimates and, if applicable, confounder-adjusted estimates and their precision (eg, 95% confidence interval). Make clear which confounders were adjusted for and why they were included	10-11
		(*b*) Report category boundaries when continuous variables were categorized	10-11
		(*c*) If relevant, consider translating estimates of relative risk into absolute risk for a meaningful time period	10-11
Other analyses	17	Report other analyses done—eg analyses of subgroups and interactions, and sensitivity analyses	10-11
**Discussion**			
Key results	18	Summarise key results with reference to study objectives	12-15
Limitations	19	Discuss limitations of the study, taking into account sources of potential bias or imprecision. Discuss both direction and magnitude of any potential bias	12-15
Interpretation	20	Give a cautious overall interpretation of results considering objectives, limitations, multiplicity of analyses, results from similar studies, and other relevant evidence	12-15
Generalisability	21	Discuss the generalisability (external validity) of the study results	12-15
**Other information**			
Funding	22	Give the source of funding and the role of the funders for the present study and, if applicable, for the original study on which the present article is based	17

*Give information separately for cases and controls in case-control studies and, if applicable, for exposed and unexposed groups in cohort and cross-sectional studies.

Note: An Explanation and Elaboration article discusses each checklist item and gives methodological background and published examples of transparent reporting. The STROBE checklist is best used in conjunction with this article (freely available on the Web sites of PLoS Medicine at http://www.plosmedicine.org/, Annals of Internal Medicine at http://www.annals.org/, and Epidemiology at http://www.epidem.com/). Information on the STROBE Initiative is available at www.strobe-statement.org.

For peer review only
